# The p53 family member p73 in the regulation of cell stress response

**DOI:** 10.1186/s13062-021-00307-5

**Published:** 2021-11-08

**Authors:** Julian M. Rozenberg, Svetlana Zvereva, Aleksandra Dalina, Igor Blatov, Ilya Zubarev, Daniil Luppov, Alexander Bessmertnyi, Alexander Romanishin, Lamak Alsoulaiman, Vadim Kumeiko, Alexander Kagansky, Gerry Melino, Carlo Ganini, Nikolai A. Barlev

**Affiliations:** 1 Cell Signaling Regulation Laboratory, Moscow Institute of Physics and Technology, Dolgoprudny, Russia; 2 School of Biomedicine, Far Eastern Federal University, Vladivostok, Russia; 3 The Engelhardt Institute of Molecular Biology, Russian Academy of Science, Moscow, Russia; 4 School of Life Sciences, Immanuel Kant Baltic Federal University, Kaliningrad, Russia; 5 HSE University, Moscow, Russia; 6 Department of Medicine, University of Rome Tor Vergata, Rome, Italy; 7 Institute of Cytology, Russian Academy of Science, Saint-Petersburg, Russia

**Keywords:** Cancer hallmarks, Tumor suppressor p53, p73

## Abstract

During oncogenesis, cells become unrestrictedly proliferative thereby altering the tissue homeostasis and resulting in subsequent hyperplasia. This process is paralleled by resumption of cell cycle, aberrant DNA repair and blunting the apoptotic program in response to DNA damage. In most human cancers these processes are associated with malfunctioning of tumor suppressor p53. Intriguingly, in some cases two other members of the p53 family of proteins, transcription factors p63 and p73, can compensate for loss of p53. Although both p63 and p73 can bind the same DNA sequences as p53 and their transcriptionally active isoforms are able to regulate the expression of p53-dependent genes, the strongest overlap with p53 functions was detected for p73. Surprisingly, unlike p53, the p73 is rarely lost or mutated in cancers. On the contrary, its inactive isoforms are often overexpressed in cancer. In this review, we discuss several lines of evidence that cancer cells develop various mechanisms to repress p73-mediated cell death. Moreover, p73 isoforms may promote cancer growth by enhancing an anti-oxidative response, the Warburg effect and by repressing senescence. Thus, we speculate that the role of p73 in tumorigenesis can be ambivalent and hence, requires new therapeutic strategies that would specifically repress the oncogenic functions of p73, while keeping its tumor suppressive properties intact.

## Background

During oncogenesis, cells typically lose their ability to repress cell cycle, repair DNA or undergo apoptosis in response to DNA damage. In the majority of human cancers this is associated with aberrant function of mutated tumor suppressor p53. Intriguingly, another member of the p53 family—transcription factor p73—binds the same DNA sequences and is able to activate transcription, which theoretically could compensate for the p53 loss. However, the p73 is rarely lost or mutated in cancers, while multiple signaling pathways can repress it’s activity or stimulate the expression of transcriptionally inactive p73 isoforms. Moreover, the p73 is often overexpressed in cancers. Thus, it is not clear if p73 is a tumor suppressor or an oncogene. In this review, we address this dilemma by analyzing roles of p73 in the regulation of DNA damage response, cell cycle and apoptosis, genome stability, metabolism and senescence.

## Introduction

The p53 family of transcription factors comprises three proteins: p53, p63 and p73. The p53 protein is involved in all aspects of cancer development and progression. While p63 and p73 generally overlap p53 in their functions, they also play their specific roles [1, 2]. While p53 is lost or mutated in about half of cancers, it is not so for p73 [3–8]. On the other hand, specific p73 isoforms are overexpressed in a variety of malignancies and determine prognosis in various types of tumor including non-small lung cancer [9–11], breast cancer [5, 12, 13], stomach and esophagus adenocarcinomas [14], gliomas [15–17] and other solid tumor types [18, 19] (induction of expression levels are in Tables 1 and 2). This contrasts with studies in mice, demonstrating that the loss of a particular p73 isoform promotes spontaneous lung adenocarcinomas and lymphomas [20].

**Table 1:** Expression levels of p73 or p73 isoforms in various cancers

Cancer	Changes of p73 isoform expression	Conclusions	References
NSLC	p73↑	Total p73 does not predict prognosis	[10]
NSLC	DNp73↑	DNp73 is associated with poor prognosis	[11]
Breast	p73↓	Upregulation is associated with lower pathological grade	[13]
Breast	p73↑ in 27%	Upregulation is association with higher pathological grade	[12]
Breast	p73↑ in 38% alpha, gamma, delta epsilon and phi isoforms detected	p73 does not have a tumor suppressor role	[5]
Esophagus	DNp73↑ in 60%	Leads to b-catenin/TCF activation	[14]
Gliomas	p73↑ ependymomas and pilocytic astrocytomas	P73 does not influence p21 and MDM2 expression	[15]
Low grade gliomas	DNp73↑, TAp73↑, DeltaEx2-3↑	DeltaEx2-3 upregulation predicts progression	[16]
High grade gliomas	TAp73↑, DNp73↓	TAp73 is upregulated in high grade gliomas	[17]
Glioblastomas	DNp73↑, TAp73↓ by PCR	13 GBM samples examined by IB	[21]
Cervical	p73↑	Higher p73 protein is associated with better survival	[22]

**Table 2:** Intracellular cancer hallmarks and p73 functions in specific cancers

Cancer	Apoptosis, cell cycle	Replicative Immortality	Genomic instability, DSB response	Altered metabolism	Role of p73 isoforms in cancer	TP73 in tumor compared to normal tissue
Non-small cell lung carcinoma (NSCLC)	[24–29]	[30, 31]		[32, 33]	[34–36]	
Lung adenocarcinomas	[27]		[20]		[37, 38]	2.95
Hepatocellular carcinoma (HCC)	[39]					
Cervix carcinoma	[40–42]			[41]	[22, 43, 44]	15.2
Melanoma	[45]			[46]		
Osteosarcoma	[47–51]		[52, 53]	[54, 55]		5.4
Glioblastoma	[56, 57]				[58, 59]	5.2
Medulloblastoma				[60, 61]	[62]	–
B-cell lymphoma	[63]	[64]	[65]		[66]	33.6
T-cell lymphoma	[67, 68]					
Acute myeloid leukemia	[69–72]					
Chronic myelogenous leukemia (CML)	[73–75]			[73]		
Breast cancer	[76–79]		[80]	[81]	[82]	1.2
Colorectal cancer	[25, 78, 83, 84]	[85–87]	[88]		[84, 89]	9.6
Esophageal adenocarcinoma	[90]		[91–93]		[94]	1.1
Thyroid cancer	[95]					
Ovarian cancer	[96, 97]				[98]	4.1
Pancreatic cancer					[99, 100]	3.15
Neuroblastoma	[26, 101–103]			[104]		
Squamous carcinoma	[105, 106]				[107, 108]	1.26

The rightmost column represents the Tp73 expression in tumor tissue (Tp73Tum) in comparison to normal tissue (Tp73Norm) as a ration log2(Tp73Tum + 1)/log2(Tp73Norm + 1) [23]

In humans, p73 loss does not provide cancer cells with a selective advantage (as in the p53 case) [109, 110], in contrast, the hypothesis is that for certain tumors p73 can be essential for growth [81]. In order for cancerous cells to form a malignant tumor they must develop multiple properties including unrestricted growth, evasion of the programmed cell death, sustained angiogenesis and tissue invasion, immune system evasion and altered metabolism along with the ability to later form metastases, which are collectively known as hallmarks of cancer [111]. p73 functions in many of these hallmarks are extensively reviewed [19, 112–116]. While uncontrolled cell cycle progression, accumulation of DNA damage and evasion of apoptosis require inhibition of the p73 activity, other hallmarks—alteration of metabolism, oncogene-induced senescence—depend on the p73 transcriptional activation. Finally, we attempt to categorize cancers based on the p73 roles with respect to the cancer hallmarks, providing the clues to how p73 affects progression of neoplasms.

## Domain structure of p73 isoforms and their functions

All p53 protein family members, including p73, have multiple isoforms resulting from alternative transcriptional start sites and alternative splicing [18, 117, 118].

All p73 isoforms can be divided into two classes based on the resulting structure. Alternative transcription start sites generate either long transcripts containing transcriptional activator (TA) at the N- terminus, resulting in TA- isoforms (TAp73), which activate transcription [119–121] or shorter transcripts driven by the intragenic promoter to generate so called DN- isoforms (DNp73), lacking transcriptional activator domain, but retaining their DNA binding domains, so their protein products act mostly as dominant-negative repressors [122–126]. However, DN- isoforms can also activate transcription [127–130].

The typical domain organization of the p53 family members includes the TA- domain followed by the DNA binding domain and the tetramerization domain [131–133]. All members of the p53 family can bind the same consensus DNA to form heterotetrameric complexes between them to regulate largely overlapping gene sets [24, 120, 134, 135].

In addition, p73 also contains the sterile alpha motif (SAM) domain that is absent in p53 and responsible for the transcriptional repression by preventing interaction with p300/CBP [136]. Several isoforms (Δex2, Δex2/3) are produced as a result of alternative splicing that lack exon 2 or exons 2 and 3, which encode the transactivation domain. Therefore, they may act as dominant negatives in respect to the full-length p73. At the C- terminus, alternative splicing generates α, β, γ, δ, ε, ζ, and η isoforms [18, 137]. Thus, exon 13 deletion produces-b (TAp73b) isoform lacking the SAM domain. Moreover, the p73 C-terminus is responsible for interactions with other transcription factors such as c-Jun, Nfkb, ATF3 and many others, thereby distinguishing p73 functions from the p53 ones [42, 47, 106, 138–140].

Since the p53 and p73 DNA binding sites overlap, the p73 can bind and activate or repress the subset of the p53 target genes that regulate cell cycle and apoptosis (p21, Bax, Fas, PUMA). Moreover, p73 is regulated by the same signaling pathways as p53, such as DNA damage response pathway (ATM/ATR/CHK1 [25]) and is repressed by an E3 ligase, MDM2 [102, 141–143]. Therefore, loss of p53 functions in cancer can be, to a certain extent, compensated by TAp73 [69, 144]. At the same time, p53 target genes can be repressed by the dominant negative DNp73 isoforms [73, 145, 146]. Both TAp73 and p53 activate the DNp73 promoter, creating a negative feedback loop [53, 147, 148].

Thereby, in human carcinogenesis transcriptionally active p73 isoforms (TAp73b) are pro-apoptotic and anti-oncogenic, while transcriptional repressors (DNp73, ΔEx2-3p73) are anti-apoptotic and pro-oncogenic [16, 21, 84, 123–126, 149, 150].

In contrast to this paradigm, both TA- and DN-p73 isoforms are frequently overexpressed in cancers [16, 21, 36, 84, 98, 123–126, 149–154] (Table 2).

Thus, pro-apoptotic p73 functions must be de-activated or bypassed in the proliferating cancer cells [25–27, 49, 57, 76, 101, 155]. Therefore, a better understanding of diverse p73 repression mechanisms in different cancers should facilitate the identification of targets for future drug development [39, 41, 45, 63, 67, 74, 77, 83, 90, 102, 156–158].

## Tumor-suppressor and oncogenic functions of TA- and DN-p73 isoforms in mice models

Animal models with complete ablation [122, 124, 159] and isoform-specific p73 deletions [20, 52, 159, 160] reveal interdependent, overlapping, and unique functions for p73 and other p53 family proteins. Mice lacking p73 have profound defects in pheromone sensory pathways, hippocampal dysgenesis, hydrocephalus, reproductive organs development as well as chronic infections and inflammation [122]. In contrast to p53-deficient mice, p73 knockout mice show no spontaneous tumorigenesis and most of the animals die within a month, while others could survive for up to 8 months [122]. However, mice with a knockout of pro-apoptotic TAp73 isoform live for 19 months whereas the lifespan of TAp73 ± mice is comparable with control (about 24 month). Both TAp73 ± and TAp73−/− spontaneously develop tumors (lung adenocarcinomas and lymphomas) [20]. In contrast, spontaneous carcinogenesis was not reported for DNp73−/− mice however, DNp73 was required for tumor formation by transformed MEFs in nude mice, highlighting the differential role of TA- and DN- p73 isoforms in carcinogenesis [52].

## p73 and hallmarks of cancer

### p73 in the regulation of DNA damage response

**Fig. 1:**
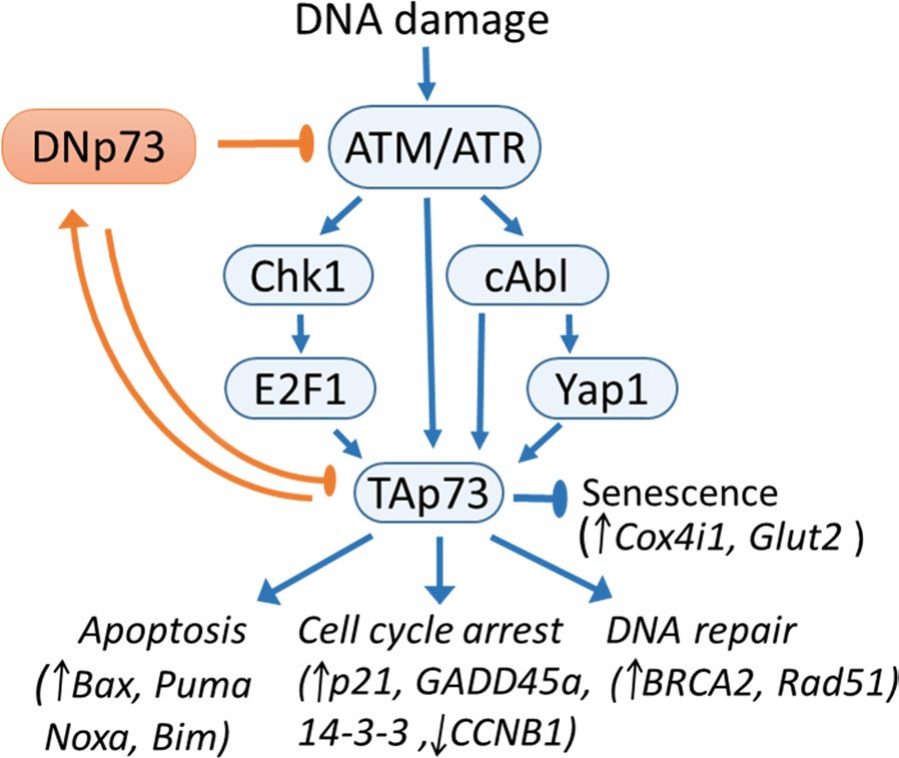
DNA damage response and cell signaling pathways that regulate p73 activity. Arrows represent activation of the pathway, and brakes highlight repression of the activity. In pink are signaling that promote and in light blue are signaling that repress p73 mediated response to the DNA damage. In orange are signaling that promote and in blue are signaling that repress cell cycle progression. A DNA damage response induces ATM/ATR/c-Abl complex formation on DNA that leads to Tyr-99 phosphorylation of p73 [161–163] or ATM/ATR mediated activation of Chk1/2-E2F1 signaling cascade [25] and Yap mediated p73 activation [164]. In turn, TAp73/p53 mediated DNp73 induction repress ATM/ATR activation [52], and repress pro-apoptotic p53/p73 responsive genes [146], providing a negative feedback loop mechanism facilitating de-activation of the pathway upon DNA repair. Activation of the p73 by DNA damage halts cell cycle progression at checkpoints

The response of the cell to DNA damage is tightly connected with the regulation of cell cycle progression. Depending on the severity of DNA damage the cell may either undergo cell cycle arrest until DNA is repaired, or senescence which permanently blocks cell cycle if DNA is unrepairable. Alternatively, cells may commit suicide by apoptosis. Importantly, cancer cells are prone to cell cycle arrest slippage and often display attenuated apoptosis. Subsequent sections will include specific p73-dependent mechanisms of the DNA damage response which regulate the decision of a cell to live to die or to senesce (Fig. 1).

Inactivating mutations or low expression of the BRCA1 gene contribute to breast and ovarian cancer development. However, BRCA1 deficient ovarian and breast tumors are sensitive to cisplatin due to induction of TAp73 [165, 166]. Mechanistically, re-expression of BRCA1 induces DNA methylation of the TAp73 promoter thereby inhibiting Zeb1 binding and Zeb1 mediated repression of the TAp73. This is in line with other investigations suggesting that DNA methylation induces tissue specific gene expression [167–169].

In contrast, the promoter of DNp73 is not methylated in the non-transformed human mammary epithelial cells (HMECs) thus permitting the inducibility of DNp73 after DNA damage and better survival after cisplatin treatment [80]. In the breast cancer cells, the level of DNp73 induction decreases with BRCA1 depletion after cisplatin treatment. Importantly, in contrast to ovarian cells, the level of TAp73 was lower in breast cancer cells [80, 165]. Interestingly, exogenous expression of DNp73 resulted in a modest, yet significant increase of cell viability after cisplatin treatment. Altogether, these findings suggest that DNp73 promotes BRCA1 deficient breast cancers [80].

Analysis of the DNA damage response (DDR) in DNp73−/− cells revealed a new role for the DNp73, as an inhibitor of the molecular signaling emanating from a DNA break to the DDR pathway [52]. Increased p53-dependent apoptosis and sensitivity to DNA damage was shown in cells from the DNp73 − / − mice [52].

Cell cycle arrest and/or apoptosis are induced by the phosphorylation of p53 by ATM [170, 171]. This process is inhibited by the DNp73, which attenuates the ATM activation. In addition, the p73 isoform DNp73b interacts with the DNA damage sensor protein 53BP1 and localizes on the sites of the DNA damage.

Overexpression of DNp73b, but not DNp73a in the γ-irradiated U2OS cells, results in interaction between 53BP1 and DNp73b, suggesting that SAM domain inhibits the interaction [52]. Furthermore, after ɣ-irradiation and in the presence of excessive amount of DNp73b, authors observed decreased ATM phosphorylation, reduced p53 protein accumulation and decreased PUMA protein expression. On the contrary, DNp73 deficiency in γ-irradiated U2OS cells stimulates attraction of 53BP1, p53, and γ-H2AX to DSB sites [52].

Isoforms of p73 control DNA damage response not only through protein–protein interactions but also via transcription of the DNA DSB repair genes [88, 93, 172–174]. For example, ɣ-irradiation of the HCT116 cells showed strong induction of the TAp73α isoform [88]. Similar to p53, the TAp73a isoform binds simultaneously with Δ133p53 to the dedicated responsive elements in the promoters of RAD51, LIG4, and RAD52 repair genes, thereby stimulating their expression [88, 175]. Furthermore, the TAp73a and Δ133p53 co-expression stimulates DNA DSB repair mechanisms, including homologous recombination (HR), non-homologous end joining (NHEJ) and single-strand annealing (SSA). Importantly, p73 knockdown results in G2 phase cell cycle arrest upon ɣ-irradiation, therefore inhibiting cell proliferation [88].

The p73 is also involved in the DNA mismatch repair pathway, via hMLH1/c-Abl/p73a/GADD45a signaling [176]. Furthermore, MMR-dependent apoptosis is blocked by specific knockdown of the p73a isoform, while G2 arrest is p73 independent.

A remarkable example of the dual role of p73 isoforms in cancer is represented by oesophageal adenocarcinoma. The latter is often caused by the gastroesophageal reflux disease (GERD). GERD is caused by the bile acid salts flux from the stomach, generating acidification, ROS and DNA damage [92]. DNA repair in the oesophageal cells is promoted by the p73. Notably, in the oesophagus, and other epithelial tissue p73 is expressed strictly in the basal cells [2]. Bile acid induces the TAp73a isoform that transcriptionally upregulates SMUG1 and MUTYH, two glycosylases which are involved in base excision repair [93]. In these conditions, TAp73α acts as a pro-survival factor that induces DNA repair enzymes and inhibits apoptosis [93], contradictory to the established pro- apoptotic functions of the wild-type TAp73 isoform discussed above [53, 147]. In turn, exposure of p53 null esophageal adenocarcinoma SK-GT-4 cells to the bile acid and pro-inflammatory cytokines IL-1β and TNFα induced DNp73α. The DNp73α induction was dependent on c-Abl, IKK, and p38 MAPK kinases [91]. In addition, the SK-GT-4 cells that exogenously expressed DNp73α showed increased survival upon bile acid exposure.

Altogether, these data highlight the pro-survival role of DN- and TA- p73 isoforms facilitating DNA repair.

### p73 in the regulation of cell cycle

#### Regulation of the G1 checkpoint

Upon DNA damage, p53 is phosphorylated by ATM/ATR/Chk1,2 and MDM2 mediated proteasomal degradation of 53 is inhibited. Alternatively, upon oxidative or oncogenic stress, p14ARF interacts with MDM2, thereby also stabilizing p53. As a result, p53 now binds DNA [177] and activates a set of genes that inhibits cell cycle progression (p21, 14–3-3σ, Gadd45). p21 inhibits CyclinD/CDK4,6 complex formation leading to Rb hypo-phosphorylation and interaction with transcription factor E2F, inhibiting cell cycle related E2F target genes leading to the growth arrest. In addition, expression of the distinct set of cell cycle related genes regulated by the p53-p21-DREAM-E2F/CHR is inhibited [178, 179].

However, even in the absence of p53, cancer cells are still able to halt cell cycle in response to genotoxic stimuli [180, 181]. It was shown that in response to chemotherapeutic agents, cell cycle arrest and apoptosis are p73 dependent [71, 156, 157, 182]. Accordingly, p63 and p73 are required for the p53 dependent apoptosis in mouse embryonic fibroblasts [183].

**Fig. 2:**
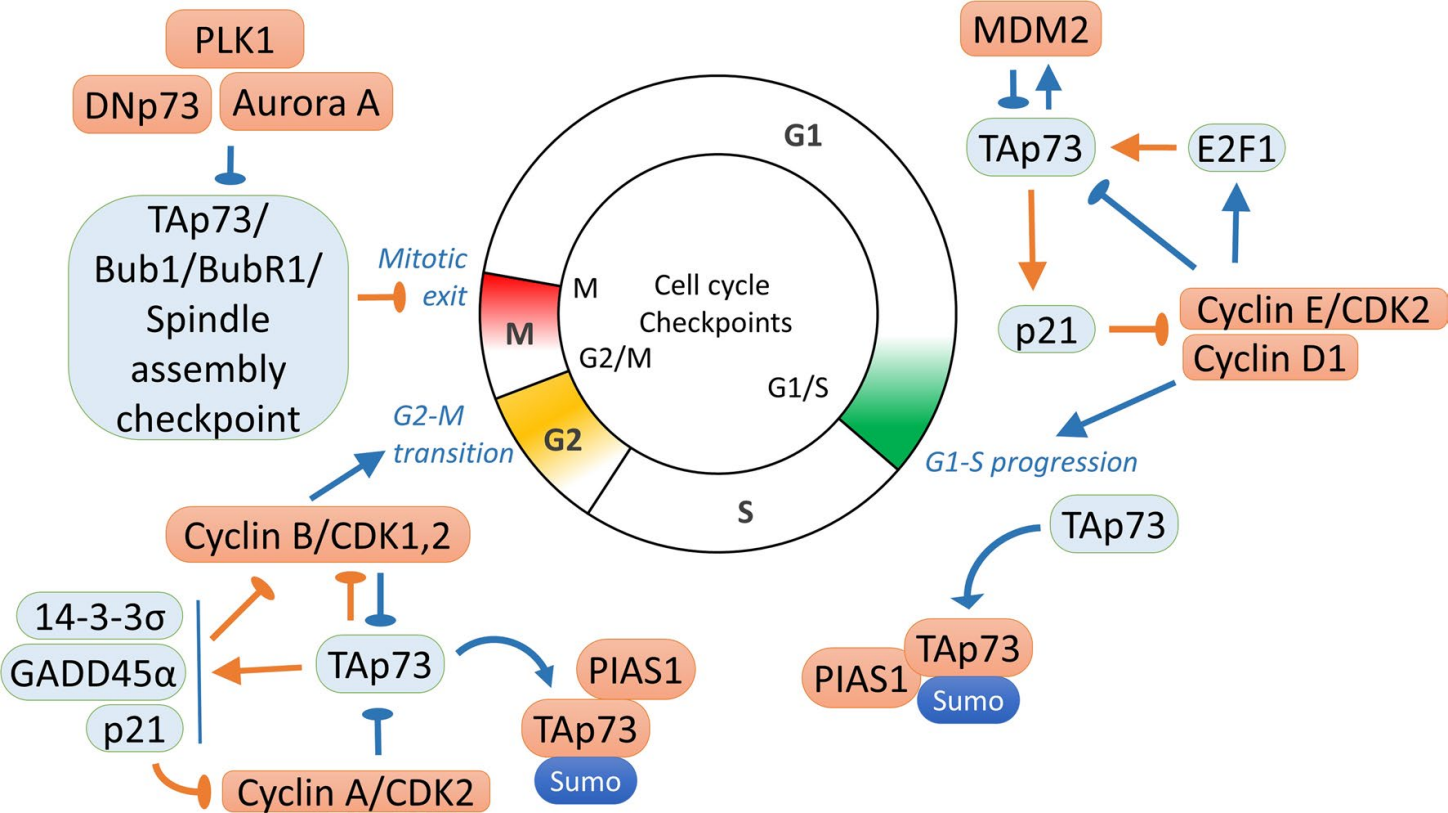
Cell cycle regulation by p73. In blue are molecules and signalling that promote cell cycle and in orange are signaling that repress cell cycle progression. Colors on the cell cycle diagram represent p73 activity that decreases at checkpoint exit [81, 186, 188]. G1/S checkpoint. Active TAp73 induces p21 leading to repression of the cell cycle related genes [70]. In turn, TAp73 transcriptional activity is inhibited by phosphorylation at Thr-86 by CyclinE/CDK2 complexes [28], or by inhibition of E2F1 transcriptional activity by the E2F1/Rb complex [189–191]. G2-S checkpoint: TAp73 inhibits Cyclin B transcription [192] or via induction of GADD45a [193], Wee, p21 and 14–3–3σ [76, 90], that represses CyclinB/CDK1 [194, 195]. To facilitate G2-M transition, p73 activity is inhibited by CyclinB/CDK1 [188], by MDM2 negative feedback loop [196] or by PLK2 [76]. Mitotic checkpoint: overexpressed TAp73 interacts with SAC components Bub1, Bub3, BubR1 leading to checkpoint activation [197, 198]. DNp73 was shown to bind to SAC proteins but have no activity in M- checkpoint [198] or to inhibit checkpoint functions [199, 200]. Phosphorylation of the p73 S235 by Aurora-A kinase results in inactivation of its DNA damage and spindle assembly checkpoint activation functions and mitotic exit due to release of the Mad2-Cdc20 from the SAC spindle assembly checkpoint complex [201, 202]

Mechanisms of the G1 checkpoint sensing by p73 and p53 are similar [184] (Fig. 2). Specifically, expression and stability of the transcriptionally active p73 is induced by the DNA damage via ATM/ATR/Chk1 pathway [25, 105, 145, 185]. Importantly, p73 is phosphorylated at Thr-86 during cell cycle progression by Cyclin E/CDK2, Cyclin A/CDK1/2, and Cyclin B/CDK1/2 complexes leading to repression of the p73b transcriptional activity [28, 186]. On the contrary, PKC dependent phosphorylation of Ser-388 [130] induces transcriptional activity of p73 towards the cell cycle regulatory genes p21, Gadd45a, Wee, 14–3–3σ causing cell cycle arrest [70, 76, 90, 187] (Fig. 2).

**Fig. 3:**
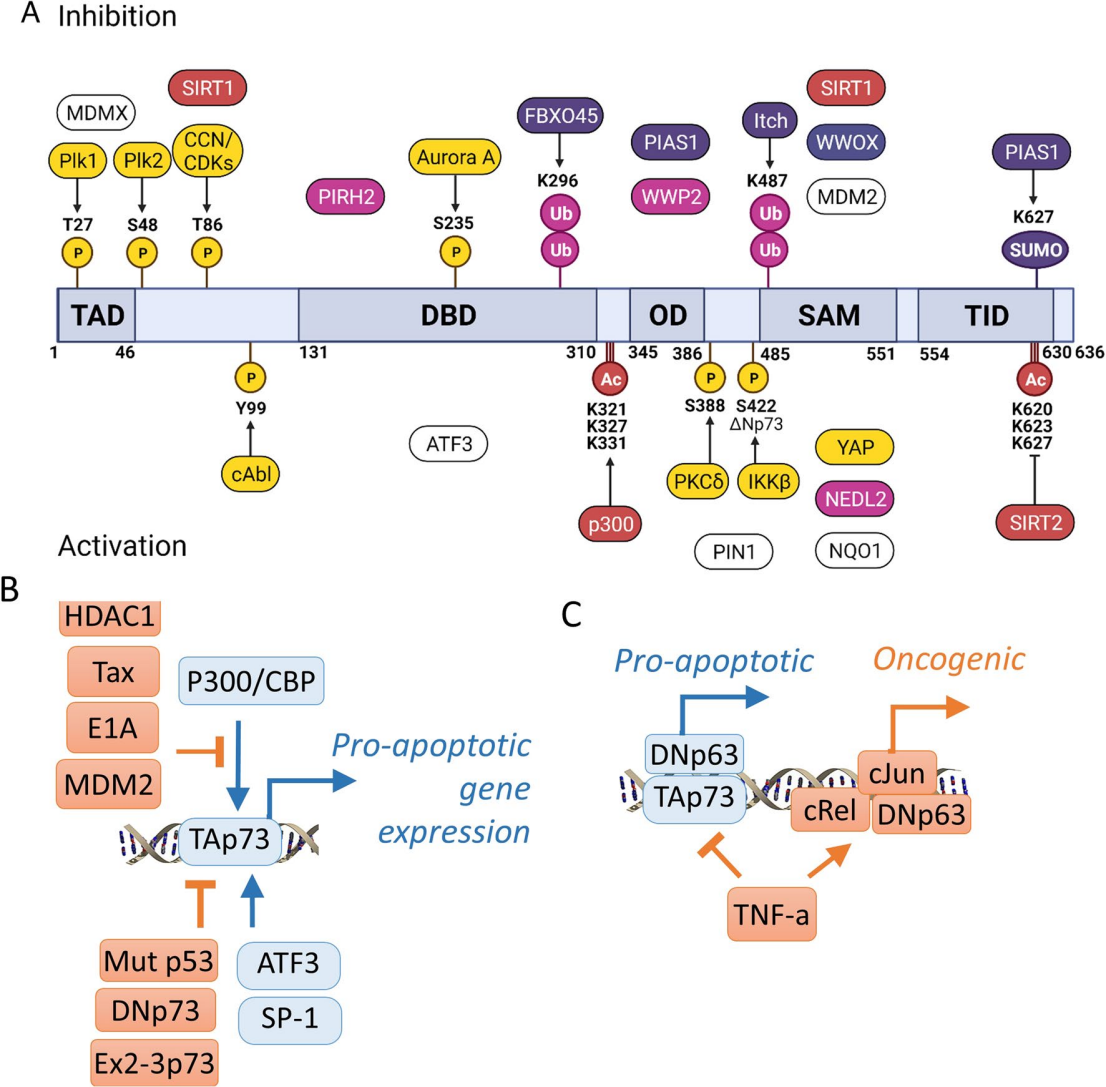
Signaling pathways that regulate p73 activity. **a** The diagram shows the TAp73ɑ isoform domain structure and known phosphorylation sites and kinases (yellow), ubiquitinylation sites and ubiquitin ligases (purple), acetylation sites (red), and the sumoylation site (violet). The positions of PTM sites correspond to the TAp73ɑ isoform unless indicated otherwise. TAD, transactivation domain; DBD, DNA binding domain; OD, oligomerization domain; SAM, sterile alpha motif; TID, transcription inhibition domain. See text for the literature. **b** Regulation of transcriptional activation. In red are denoted proteins and signalling that inhibit transcriptional activation of TAp73 [56, 79, 102, 123, 126, 235, 236] and in blue are signalling pathways and proteins that promote TAp73 regulated pro-apoptotic gene expression [42, 237, 238]. **c** In squamous carcinoma, TNFa induces nuclear translocation of cREL and induces re-localization of TAp73 binding sites from pro-apoptotic to pro-oncogenic [140]

What are the mechanisms that inhibit p73 mediated cell cycle arrest in normal and cancer cells? Numerous signaling pathways regulate p73 transcription, translation, stability and cell cycle progression [56, 130, 184, 203–205] and apoptosis [25, 29, 42, 50, 51, 75, 206–211] (Fig. 3a).

Specifically, negative regulators include E3 ubiquitin ligases Itch, Pirh2, FBXO45, WWP2 and SUMO ligase PIAS1 which promote p73 degradation in 26 proteasomes [212–217] (Fig. 3a).

Binding p73 to ATF3 prevents the ubiquitination and degradation of p73 [42]. Unlike the other E3 ubiquitin ligases, ubiquitinylation by NEDL2 enhances p73ɑ stability, while the interaction with NQO1 protects p73ɑ from the ubiquitin-independent degradation in 20S proteasome. Notably, NEDL2 regulates metaphase to anaphase transition [218, 219] (Fig. 3a).

The p73 transcriptional activity is induced by PKC [130, 220] and c-Abl [221] phosphorylation and YAP1 mediated corecruitment of p300 [222, 223]. Upon c-Abl-mediated phosphorylation, the prolyl isomerase Pin1 induces conformational changes of p73 required for its acetylation by p300 [224] (Fig. 3a).

SIRT2, a NAD-dependent histone deacetylase, is required for glioblastoma stem cells proliferation and tumorigenicity [56]. Furthermore, SIRT2 regulates p73 transcriptional activity by deacetylating its C‐terminal lysines 620, 623, and 627. Importantly p73 inactivation (in the absence of p53) by SIRT2 is critical for glioblastoma cells proliferation and tumorigenicity [56]. In HEK293 cells, SIRT1 binds p73 in vivo and in vitro, deacetylates it, and suppresses p73‐dependent transcriptional activity thereby partially inhibiting p73-dependent apoptosis [225].

The IKKβ protein kinase, which regulates NFkB, also binds DNp73a and phosphorylates the latter on Ser-422, causing p73 stabilization, nuclear accumulation and repression of the p53-regulated genes in several cancer cell lines [226, 227].

In contrast, in p53-null squamous cell carcinoma cells, TNF-α promoted c-REL nuclear translocation, c-REL/DNp63α interaction, and TAp73 dissociation from DNp63α. This chain of events culminates in TAp73 translocation from the nucleus to cytoplasm [106]. TNF-α modulates genome-wide redistribution of DNp63α/TAp73 and NF-κB c-REL cumulative binding at the TP53 and AP-1 DNA binding sites to induce an oncogenic gene expression program in squamous cancer [140] (Fig. 3c).

On the related note, the TAp73 can inhibit wild type p53 activity and induction of apoptosis [97, 228]. Similarly, the complex of p53 with chaperones and MDM2 deactivates TAp73 [229] (Fig. 3b). In contrast, p53/TAp73 can activate each other's functions in different cells by MDM2 quenching [95] or co-recruitment upon p53 Thr-81 phosphorylation [88, 230] while other demonstraetd that Mdm2 and MdmX binding represses p73 masking its transcription [217].

Viral infection causes development of cancers such as cervical and Kaposi sarcoma [231]. The activity of p53 family members in viral oncogenesis has been established [232, 233]. Viral onco-proteins were shown to fine-tune the p73 activity. For example, interaction of p53 and p73 with CBP can be inhibited by the human T-cell leukemia virus type1 Tax protein [68] as well as by adenovirus E1A protein [234].

Thus, the effects of p73 isoforms on the cell cycle and apoptosis are highly diverse and different mechanisms in turn are utilized in cancers to repress the p73 activity.

#### p73 mediated regulation of replicative G2-M checkpoint

Upon completion of the DNA synthesis, cells execute G2/M replicative checkpoint. During this stage, DNA integrity is verified and repaired as well as the levels of proteins required for the mitosis are evaluated.

The p73 is involved in the regulation of the DNA damage response during G2-M and M checkpoints [28, 41, 81, 88, 90, 188, 216, 239] (Fig. 2). The ability of cells to activate p73 correlates with the specific stage of the cell cycle. Specifically, the activity of p73 is high at the checkpoints and low upon checkpoints exit, regulating the key cell cycle related genes [81, 186, 188] (Fig. 2). For example, a p73/SP1 heterodimer represses CyclinB1 transcription [192]. In turn, there is a negative regulation of p73 by CyclinB1/CDK1 complex that associates with and phosphorylates p73 at Thr-86 resulting in the inhibition of DNA binding and transcriptional activity of p73 upon progression from the G2 phase to mitosis [188]. c-Abl kinase interacts with ATM/ATR participating in DNA damage Chk1 phosphorylation, p53 and p73 activation [161–163]. c-Abl phosphorylates p73 at Tyr99 leading to inhibition of p73 degradation by TRIM28 E3 ligase, subsequent p73 stabilization and activation of the anti-oncogenic program [221] A similar stabilization mechanism has also been described in the context of pharmacological stabilization of p53 [240]. It has been suggested that c-Abl/p73 pathway is active during G2/S checkpoint because at G1 checkpoint hypophosphorylated Rb inhibits E2F1 and c-Abl [190, 241]. Another mechanism of G1/S and G2/M exit is mediated via inactivation of p73 by PIAS-1 binding that stabilises and symoylates p73-alpha isoforms inhibiting transcriptional activity. Importantly, PIAS-1 is expressed exclusively in the S-phase and PIAS-1 knockdown leads to accumulation of cells in the G2 phase of cell cycle [216].

#### Genome stability and p73 in regulation of mitosis. M checkpoint

Errors in the molecular mechanisms responsible for regulation of the chromosome segregation during meiosis and mitosis decrease the genomic stability, which leads to aneuploidy. The M checkpoint occurs between metaphase and anaphase, right before the onset of chromosomal separation to ensure their proper alignment. To execute the “M” checkpoint, cells assemble a so-called spindle assembly checkpoint complex (SAC) that is localized at the kinetochores and measures correct attachment of microtubules to the spindle poles [242, 243]. This complex activates the mitotic checkpoint complex (MCC) that in turn binds to and inhibits activity of the Anaphase promoting complex (APC/Cdc20) which activation leads to the cohesin cleavage, segregation of chromosomes (mitotic exit) and degradation of the CyclinB1 [244–247].

A role of the p73 in regulation of genomic stability during mitosis has been extensively characterized [20, 28, 104, 186, 188, 198, 199, 201, 202, 248] (Fig. 2).

During the drug induced M-phase arrest of Hct116(3) cells, the TAp73 is phosphorylated by the Cdc2-CyclinB, losing the DNA binding capacity and transcriptional activity [188]. During mitosis, the p73 was not associated with the centromeres and was excluded from the condensed chromosomes in H1299 cells [188]. However, the TAp73-deficient mouse embryonic fibroblasts (MEFs) were severely compromised in undergoing a nocodazole-induced mitotic arrest [20]. Furthermore, TAp73 − / − MEFs underwent a premature mitotic exit and passage into the G1 phase, suggesting that mitotic regulation is predominantly performed by the TAp73 isoforms rather than DNp73 isoforms [20].

Treatment of TAp73 − / − lung fibroblasts with nocodazole for 12 h resulted in the increased polyploidy as well, whereas TAp73 − / − thymic cells showed no alternations in genomic stability [20]. This suggests that the effect of TAp73 on the aneuploidy is tissue-specific, which could be an explanation to why TAp73 − / − mice preferentially develop lung adenocarcinomas [20].

There is multiple evidence to suggest that when overexpressed, TAp73 interacts with SAC components Bub1, Bub3, BubR1 leading to M checkpoint activation [197, 198]. In triple-negative breast cancer MDA-MD231 cell lines, endogenous TAp73 isoform interacts with BubR1, whereas DNp73 is not [198]. Upon overexpression, DNp73b does interact with BubR1, however, in contrast to TAp73a, its binding does not affect the interaction of BubR1 with phosphorylated histone H1 suggesting that DNp73 does not have a role in SAC function [198]. In contrast, overexpression of DNp73b leads to tetraploidy in human lung carcinoma H1299 cells [199] suggesting that DNp73 affects genomic stability as well. Accordingly, in glioblastoma cells DNp73 isoform overexpression leaded to an abnormal number of centrosomes, while TAp73 overexpressing cells showed normal centromeres count with no association with BubR1, suggesting that DNp73 can inhibit checkpoint activation [200].

In contrast, in Saos2 osteosarcoma cell line, TAp73α overexpression led to a significant increase in the number of polyploid cells, while p53, DNp73α, and TAp73β or γ had no effect on polyploidy [197]. The effect was linked to the interaction of TAp73α with Bub1 and Bub3 that were also overexpressed in these cells whereas neither p53 nor any of the other p73 isoforms interacted with Bub1 or Bub3.

Phosphorylation of the p73 S235 by Aurora-A kinase results in inactivation of its DNA damage and spindle assembly checkpoint activation functions and mitotic exit is accelerated due to release of the Mad2-Cdc20 from the SAC spindle assembly checkpoint complex [201, 202]. In addition, inhibitor of Aurora kinase B AZD1152-HQPA induces apoptosis in p53/p73 wild type U87MG glioblastoma cell line, whereas in p53/p73 double null SK-N-MC cells inhibition of Aurora kinase B leads to induction of cell cycle related genes, endoreplication and polyploidy, highlighting the role of p53/p73 [104, 201].

All together, these publications reveal the roles of TAp73 as an activator and DNp73 as an inhibitor of SAC activity and, correspondingly, TAp73 as an inhibitor and DNp73 as an activator of early mitotic exit and chromosomal abnormalities.

DNA damage can occur at any stage of the cell cycle and p73-activated checkpoints are utilised by cells for DNA repair and maintaining genome stability. If DNA damage or SAC signals persist, the p53/p73 activates transcription of pro-apoptotic genes, whose products ultimately trigger apoptosis [249].

### Regulation of apoptosis by p73

Apoptosis is mediated by dynamic equilibrium of activating and repressing signals. A key event of triggering apoptosis is accumulation of the cytoplasmic protein Bax on the outer mitochondrial membrane [250]. Bax oligomerization and formation of pores on the outer mitochondrial membrane leads to the release of cytochrome C in the cytoplasm, apoptosome assembly and subsequent caspase-mediated cleavage of cellular proteins [251]. At the late stages, apoptosis culminates in DNA fragmentation [252] and induction of phagocytosis [253].

On the other hand, Bax mediated release of the cytochrome C is repressed by Bcl-2 protein family (Bcl-2, Bcl-XL, Mcl-1, Bcl-w, A1, Bcl-L10, Bcl-G and Bcl-Rambo) whereas, so called BH3-only family of proteins (such as Puma, Noxa, Bad, Bid, Bim, Hrk, Bik and Bmf) bind to and inhibit the Bcl-2 anti-apoptotic functions [254].

**Fig. 4:**
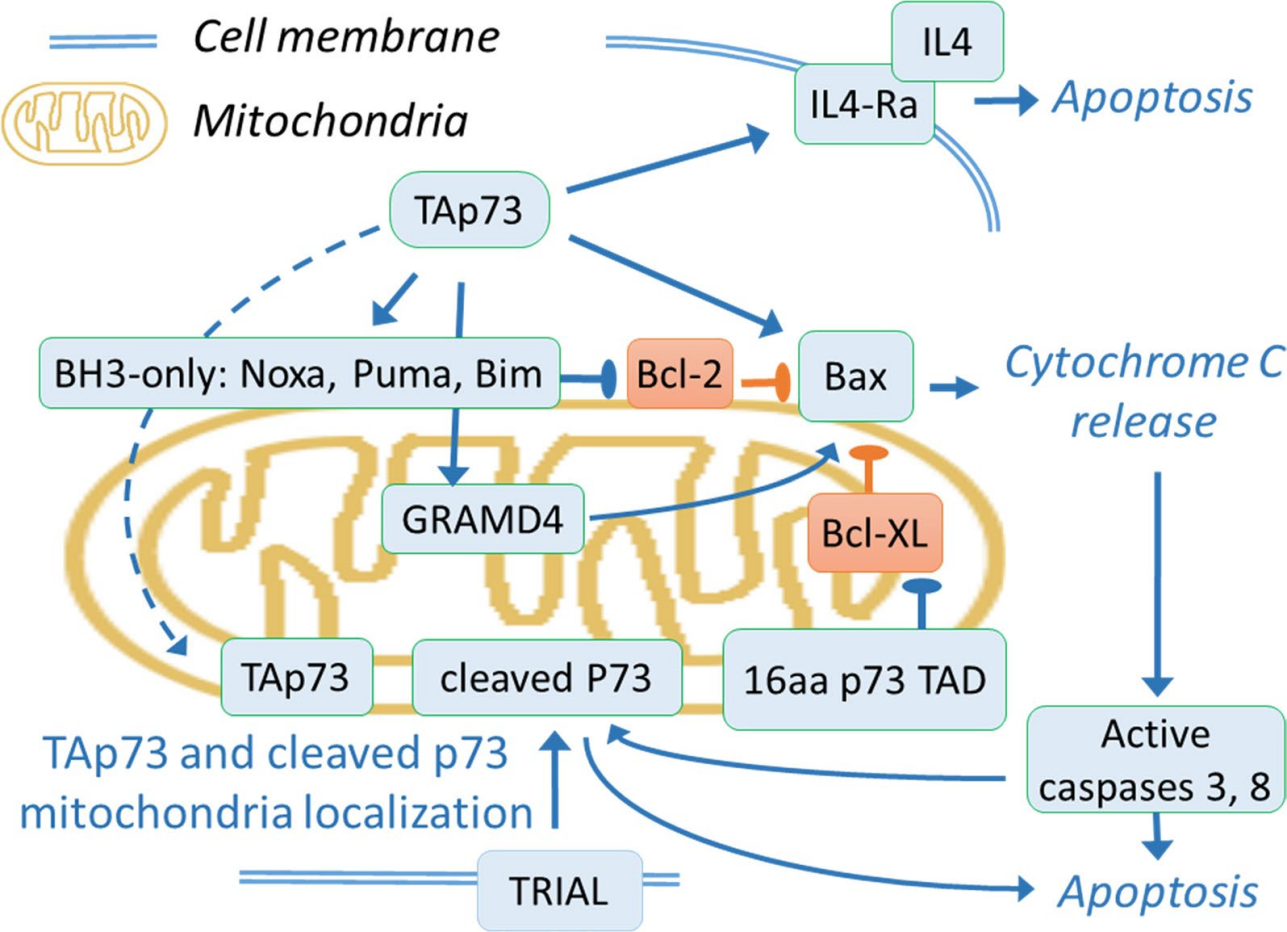
Schematic representation of p73 involvement in the regulation of apoptosis. In blue, molecules that promote and in orange—molecules that repress apoptosis. Arrows and bars in green—signalling that promote and in red—repress apoptosis and senescence. A. p73 mediated regulation of apoptosis. Transcriptional activation of p73 promotes expression of Bax [41, 255–259] and BH3-only family members PUMA [228, 260–263], Noxa [238], Bim [248] and GRAMD4 [264] that repress Bcl-2 and induces Bax activity. The p73 regulated induction of IL4R sensitize cells to IL-4-induced apoptosis [265]. In turn, p73 repressing pathways inhibit apoptosis [266]. Alternatively, p73 or p73 fragments can localize to mitochondria during TRIAL induced apoptosis [267] and 16 aa peptide from the transactivation domain of p73 can interact with and inhibit Bcl-x [268]

Specifically, p73 is required for induction of apoptosis by regulating transcription of key pro-apoptotic genes (Fig. 4). Transcriptional induction of Bax by p73 leads to apoptosis in different settings [41, 255–259]. In addition, mechanisms of p73 induction of apoptosis involve transcriptional activation of PUMA which, in turn, induces Bax mitochondrial accumulation and release of cytochrome C [228, 260–262]. The p73 phosphorylated by Aurora-A at S235 loses the chromatin binding affinity, and is exported from the nucleus to cytoplasm [201], thereby halting the expression of its transcriptional targets such as p21 [201] and pro-apoptotic BH3-only protein Bim. This affects cytochrome C release and caspase activation [248].

Similarly, p73 binds to the promoter and transcriptionally activates GRAMD4. As a result, apoptosis is induced via GRAMD4 translocation from nucleus to mitochondria and interaction with Bcl-2 [264].

It was shown that p73 could mediate cell death independently of other p53 family members. In the p53 deficient cells, p73 is induced upon DNA damage by E2F1 transcriptional activation downstream of Chk1/2 [25, 269–271] (Fig. 1). However, as it is discussed in the previous section, effects of p73 are dependent on other p53 family members' activity. For example, the loss of p63 in normal keratinocytes causes p21 induction, inhibition of cell cycle and senescence independent of p53 and p73, whereas in squamous cell carcinoma the loss of p63 induces p73-dependent cell death [272]. Accordingly, in head and neck squamous cell carcinoma cells, the p63 knockdown induces pro-apoptotic Puma and Noxa and cell death in p53 independent and p73-dependent manner [273]. Similarly, in triple negative breast cancer cells, p63 inhibits p73 activity and apoptosis mediated by PUMA and NOXA [274]. In several human cancer cell lines, interleukin 4 receptor alpha is up-regulated by p73 but not significantly by p53, sensitizing cells to IL-4-induced apoptosis [265]. FBXO45 binds specifically to, and ubiquitylates p73 triggering its proteasome-dependent degradation. When FBXO45 is downregulated, p73 is stabilized and it consequently induces cell death in the p53 independent manner [214].

In addition, p73 apparently regulates apoptosis in a transcription-independent manner (Fig. 4). Intriguingly, caspase cleaved p73 fragments augment TRAIL induced apoptosis of HCT116 cells independently of p73-mediated transcription [267]. Accordingly, in lung adenocarcinoma cells, a peptide from the p73 transactivation domain interacts with Bcl-XL and mediates transcription-independent apoptosis [268]. In turn, scaffolding protein RanBP9 interacts with, and stabilises p73a, inducing mitochondrial dysfunction and apoptosis in the primary hippocampal neurons by transcriptional effects and likely directly at mitochondria [275].

An important aspect of cancer cells is their ability to switch between cell cycle arrest and apoptosis in the cases of irreparable damage. Interestingly, p73 differentially activates cell cycle related and pro-apoptotic genes [130, 190, 228, 257, 276, 277].

Differential p73 effects on cell proliferation and apoptosis upon doxorubicin treatment were dependent on specific post-translational modifications. For example, in osteosarcoma cancer cells and mouse embryonic fibroblasts p300 in cooperation with c-Abl enhanced p73 acetylation at lysines 321, 327, and 331 [276, 278]. Accordingly, in the chronic myeloid leukemia cells, forced nuclear localization of the Bcr-Abl fusion protein promotes p73 activation and subsequent apoptosis [279].

In p53-/- lung adenocarcinoma cells, overexpression of TAp73a increases apoptosis induced by the cisplatin treatment, which was inhibited by E3 ubiquitin ligase CHIP overexpression. This effect was determined by the binding of CHIP to the C-terminus of p73a isoforms and targeting of the p73 to proteasomal degradation [280]. Notably, overexpression of CHIP has no effect on p73b isoforms [280].

In contrast, in colon cancer cells with functional p53, TAp73 restrained p53 mediated activation of apoptosis after low levels of DNA damage by forming a protein complex with p53 that is incapable of DNA binding at Puma, p21 and Bax promoters [228]. Interestingly, the p73 was induced and complexed with the p53 in cells treated with small doses of cisplatin, awhile after higher cisplatin doses, p73 induction and high molecular weight p53 complex were not observed, facilitating activation of the pro- apoptotic genes [228].

Although there are no publications describing the direct effect of p73 on necroptosis and ferroptosis, as opposed to p53 and p63, it is tempting to speculate that p73 acts as a repressor of ferroptosis since p73 regulates genes such as glucose-6-phosphate dehydrogenase, a rate-limiting enzyme of the pentose phosphate pathway [32, 55, 281–283]. The product of this gene is pivotal for glutathione biosynthesis and anti-oxidative cellular response [284].

Thus, p73 functions as a part of the p53 signaling pathway and its overall effects are modulated by the p53 status and cell type [285]. Also, p73 plays a unique role in the regulation of cell cycle and apoptosis [286, 287].

### p73 dependent regulation of metabolism

Consistent with the hallmarks of cancer defined by Weinberg [111] the metabolic alterations in cancer include:

1. Utilization of glycolysis for energy production in hypoxia or in the presence of oxygen (so called Warburg effect) with lactate production and acidification of extracellular environment and enhanced biosynthesis [288, 289].
2. Increase of the reduced glutathione production leads to chemoresistance and lower sensitivity to superoxide radicals produced by immune cells [116, 290].

In general, metabolic alterations can be traced back to crucial molecular events such as mutations in critical genes, building gene associated gene signatures [291, 292].

In this context, the role of p73 in metabolism was recently reviewed in an excellent publication [116] and here we will discuss only the cancer-related findings.

**Fig. 5:**
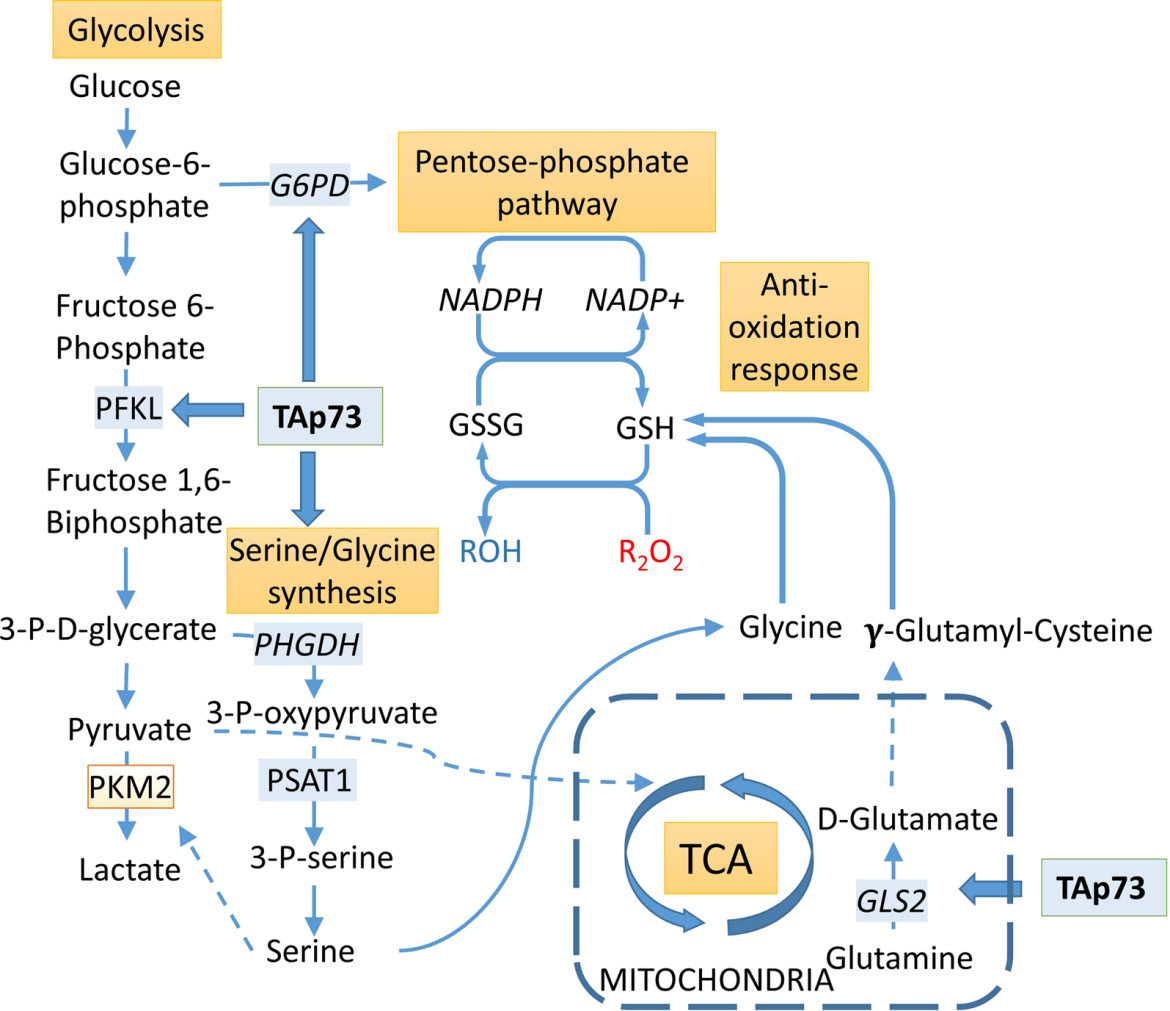
Metabolic signaling regulated by p73. The p73 regulates expression of glucose-6-phosphate dehydrogenase, inducing pentose phosphate pathway and anaerobic glycolysis [32, 55, 281, 282] glutathione production and response to oxidative stress and xenobiotics [297–300]. Further down the glycolysis pathway, TAp73 transcriptionally regulates liver type phosphofructokinase-1 (PFKL) that enhances glycolysis rate [33] and enzymes of the serine/glycine biosynthesis [54] necessary for cell growth in serine deprivation and regulation of pyruvate kinase (PMK2) which activity is associated with lactate production and cancerogenesis [304, 305]. Further, p73 regulates expression of the glutaminase-2, which converts glutamine to glutamate influencing glutathione synthesis [54]

The p73 is known to regulate several key enzymes of glycolysis and energy production [33, 293], anabolism of amino acids and detoxification [54, 294–296] (Fig. 5). Importantly, p73 influences expression of glucose-6-phosphate dehydrogenase (G6PD), a rate-limiting enzyme of the pentose phosphate pathway (PPP) [32, 55, 281, 282]. The fraction of PPP in glucose consumption is about 10% and is rapidly increasing upon oxidative stress to produce NADPH—a metabolite necessary for generation of the reduced glutathione—the main ROS and xenobiotics scavenger of the cell [32, 33, 55, 297–302]. Altogether, this increasing amount of evidence is also showing the potential role of oxidative stress as a candidate therapy target [303].

TAp73 overexpression in Saos-2 cells was shown to enhance the Warburg effect by inducing the level of lactate produced during glycolysis. Concomitantly, these cells exhibited a decreased level of pyruvate but increased levels of acetyl-CoA, S-adenosylmethionine, and cysteine [294]. Cellular anabolism is also influenced by the ability of TAp73 to stimulate transcription of serine biosynthesis enzymes and glutaminase-2, which converts glutamine to glutamate [54]. Importantly, lung cancer patients with coordinately increased p73 and GLS-2 were characterized by significantly worse prognosis [54]. In addition, enhanced serine production activates PMK2 and promotes conversion of pyruvate to lactate [304].

Thus, TAp73 overexpression promotes production of metabolic intermediates cysteine and glutamate that are converted by glutamate-cysteine ligase to glutathione, thereby enhancing the antioxidant defense.

In E1A/H-RasV12-transformed mouse embryonic fibroblasts, TAp73 is crucial for the G6PD transcription, and consequently for PPP functioning, NADPH homeostasis, cellular growth and tumor formation [55]. Notably, depletion of TAp73 was rescued by G6PD expression or supplementation with nucleosides and ROS scavenger in MEFs [55]. Similarly, experiments on H1299 non-small lung carcinoma cells showed that p73-depleted H1299 cells overexpressing G6PD grew the same as control, proving that G6PD expression is vital for TAp73-mediated H1299 cells proliferation [32].

Similarly, in E1A/H-RasV12-transformed mouse embryonic fibroblasts, osteosarcoma U2OS, and lung cancer H1299 cell lines TAp73 transcriptionally regulates phosphofructokinase-1, liver type (PFKL) and promotes glycolysis [33]. Notably, decreased proliferation of TAp73^−\−^ MEFs or tumorigenicity of HCT116 cells was rescued by PFKL and PFKL/G6PD overexpression respectively [33].

The p73 is often overexpressed in medulloblastomas. Furthermore, TAp73 appears to be critical for the medulloblastoma cells proliferation as its expression correlates with glutamine level. Accordingly, it was shown that glutamine starvation along with injections of cisplatin led to increased apoptosis in both in vitro and in vivo experiments on medulloblastoma xenograft mice [60, 61].

To summarize, TAp73 promotes cancerous metabolism, oxidative and xenobiotic stress response, and the Warburg effect both in in vitro experiments and mice models, all together supporting tumor growth.

### Senescence and replicative immortality

Telomerase is a ribonucleoprotein that synthesizes telomere repeats at the DNA ends in embryonic tissue, in germline tissue and in the immortalized cancer cell lines. In the normal somatic tissue, telomerase is not active [306] and hence, with increasing number of cellular divisions the telomeres repeats are gradually lost. This mechanism limits the replicative potential of the cell, thereby preventing replicative immortality.

Senescence is defined as a permanent growth arrest of the metabolically active cell without cell death associated with the telomere shortening after about 40 cellular divisions [307–310].

In addition, normal cells preferentially undergo senescence rather than cell death in response to various forms of stresses including the oncogene activation [311, 312], DNA damage [313] and oxidative stress [314–317].

Senescence is deemed as an anti-cancer cellular response that compliments apoptosis [318] in response to sub-lethal doses of stress [319, 320].

The senescence signaling pathways are frequently altered in cancers [321, 322]. This is exemplified by the mutations in key senescence genes such as p53, CDKN2A, CDKN2B, TERT promoter [321].

On the molecular level, senescence is mediated by activation of p14ARF that interacts with MDM2, hence preventing p53 degradation and eliciting the growth arrest. Independently, p16INK inhibits the CyclinD1/CDK4/6 complex leading to the RB hypo-phosphorylation and sequestering the transcription factor E2F. The latter event results in transcriptional inactivation of cell cycle-related E2F target genes and subsequent growth arrest, which can be rescued by the alteration of other molecular events in specific cancer contexts [323].

Notably, senescence regulation differs in mice and humans [324, 325]. While mice fibroblasts require p14ARF-p53 for oncogene induced senescence [324, 326, 327], human cells are p53 independent and in turn require p16INK-CyclinD/CDK4/6 signal transduction [324, 325]. Of note, p53 mutations in cancer cells contribute to bypassing senescence in response to oxidative stress [315].

**Fig. 6:**
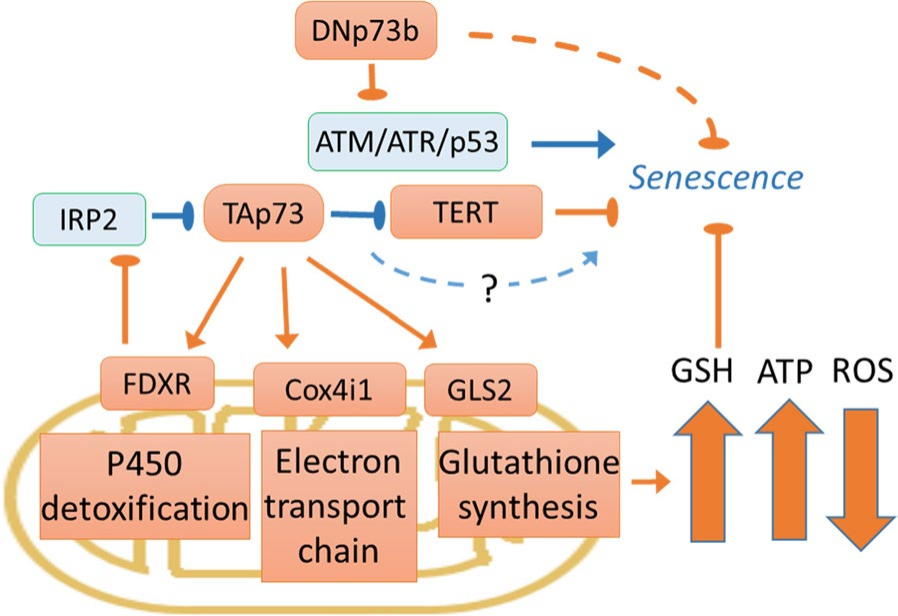
Schematic representation of p73 involvement in the regulation of senescence. In blue, molecules that promote and in orange—molecules that repress senescence. TAp73 inhibits TERT activity, however, the effect on senescence is not established [329]. In contrast, DNp73 represses p53/p73 activating signalling emanating from the DNA double stranded breaks by the ATM/ATR signalling also repressing senescence [52]. TAp73 induced transcription of the mitochondrial enzymes Cox4i1 regulating ROS, ATP levels and oxygen consumption is critical for senescence repression in MEFs [87]. Similarly p73 target genes GLS2 is a key enzyme in glutathione biosynthesis to reduce ROS level [330, 331] and FDXR has roles in electron transfer to p450 and p73 mRNA stability by IRP2 regulation [332]. Altogether, these processes lead to higher oxidation rate and ATP generation, glutathione biosynthesis, lower ROS levels and inhibition of senescence

As it is discussed in the corresponding sections of our review, p73 is involved in the regulation of the DNA damage response, metabolism and oxidative stress responses [30, 31, 290, 328] thereby influencing senescence (Fig. 6).

While the role of the p53 family proteins in regulation of cellular senescence is firmly established [85, 322, 333–338], several publications also demonstrate the role of p73 in this process [52, 64, 87, 329, 333, 339–341] (Fig. 6).

Telomeres are protected from the DNA damage by a protein complex called shelterin [86, 342]. If the shelterin component Pot1b is depleted in p63 knockout mice, telomeres activate ATR-Chk1 DNA damage response and p73 dependent apoptosis [64].

It was shown that overexpression of the TAp73a or TAp73b isoforms represses TERT transcription and TERT activity in the HEK cell line [329]. Using luciferase reporter assays, it was shown that p73-mediated TERT reporter repression was rescued by the NF-YB depletion. The TERT transcription and TERT activity were also repressed by the overexpression of TAp63g and p53 [329]. Even though senescence was not examined, the TAp73 overexpression-mediated repression of the TERT might promote senescence of the HEK cell line [329].

However, direct experiments revealed that both TA- and DN-p73 isoforms repress senescence. Such as, E1A/RasV12 transformed MEFs generated from the DNp73 knockout mice formed smaller xenograft tumors with higher b-gal staining and higher expression of senescence markers p16 and DcR2 [52].

When TAp73 knockout mice were examined, MEFs grew slowly, and 3.5 times higher senescence levels were observed [87]. Accordingly, the p16 and p19 senescence markers were induced. Mechanistically, the effect was attributable to the inhibition of the mitochondrial complex IV activity, specifically due to the depletion of the direct TAp73 target, Cox4i1. Authors observed higher ROS level, lower ATP and oxygen consumption and higher oxidative stress sensitivity. Depletion and restoration of the Cox4i1 level mimics and inhibits effects of the TAp73 depletion, respectively [87].

Reduction of TAp73 is responsible for ROS accumulation upon depletion or knockout of the RNA-binding protein PCBP2 in H1299 cells [31], presumably through regulation of the p73—induced gene, glutaminase 2 [330], which is involved in the anti- oxidative response [331]. Although senescence was not reported in the human cell lines in that study, about the 24-fold induction of b-gal staining was observed in PCBP2 − / − MEF cells [31].

Depletion of both p73 and ferredoxin reductase (FDXR, the mitochondrial electron transfer protein) induces senescence of MEFs [332]. The p73 is reduced upon the loss of the FDXR due to induction of the iron-regulatory protein 2 (IRP2) which binds to and destabilises p73 mRNA [332]. Interestingly, FDXR is a p73 transcriptional target [343], suggesting a possible positive feedback loop between FDXR and p73. It would be interesting to compare the effect of p73 and p53 on IRP2 since restoration of decreased p53 in FDXR KO cells reduces IRP2 [344].

Consistently, induction of senescence was observed in the neural precursor cells of the p63 and p73 haplo-insufficient mice [341]. Increased DNA damage and p53 activation were responsible for the phenotype since genetic ablation of the p53 completely inhibited this effect.

To summarize, in most cases, either TA- or DN-isoforms of p73 act as senescence inhibitors. Thus, induction of p73 activity will presumably lead to either cell cycle arrest or apoptosis but not senescence, because of p73 being a positive regulator of the antioxidation metabolism.

## Conclusions

The p53 protein family members, p53 and p73, share the same DNA binding site, have similar domain structure and overlapping signaling pathways that regulate their activity. Hence, it is a long-standing question: why p73 is rarely mutated or lost in cancer while p53 aberrations are found in the vast majority of cancers and considered to be fundamental for cancer development? Evidence presented in this review suggests a dual role of the p73 in cancer. First, it is involved in the cell cycle arrest and anti-oxidative response thereby promoting cell survival. On the other hand, being a homolog of p53, it promotes DNA damage induced cell death. Finally, it can also serve as biomarker, as p53, in many cancer contexts [345, 346]. Consequently, cancer cells seem to develop mechanisms that favor pro- oncogenic and repress anti- oncogenic functions of the p73, especially in the absence of functional p53. However, understanding of signaling pathways that fine tune the balance is very limited. Hence, the exact role of TAp73 in tumorigenesis and aggressiveness remains debated, highlighting the need for further and more precise investigations. New findings on the molecular mechanisms that define the exact outcome of p73 activity in tumour cells will help to develop treatments that will specifically induce pro-apoptotic functions of the p73 in cancer.

## Data Availability

Not applicable.

## Abbreviations

HMECs: Human mammary epithelial cells
DDR: DNA damage response
TAD: Transactivation domain
DBD: DNA binding domain
OD: Oligomerization domain
SAM: Sterile alpha motif
TID: Transcription inhibition domain
SAC: Spindle assembly checkpoint
MCC: Mitotic checkpoint complex
APC: Anaphase promoting complex
MEFs: Mouse embryonic fibroblasts
PPP: Pentose phosphate pathway
ROS: Reactive oxygen species

## References

[1] Wildung M, Esser TU, Grausam KB, Wiedwald C, Volceanov-Hahn L, Riedel D (2019). Transcription factor TAp73 and microRNA-449 complement each other to support multiciliogenesis. Cell Death Differ.

[2] Rozenberg JM, Rogovaya OS, Melino G, Barlev NA, Kagansky A. Distinct p63 and p73 protein interactions predict specific functions in mRNA splicing and polyploidy control in Epithelia. Cells. 2020;10(1). 10.3390/cells10010025 PMC7824480 33375680

[3] Ikawa S, Nakagawara A, Ikawa Y (1999). p53 family genes: structural comparison, expression and mutation. Cell Death Differ.

[4] Alonso ME, Bello MJ, Lomas J, Gonzalez-Gomez P, Arjona D, De Campos JM (2001). Absence of mutation of the p73 gene in astrocytic neoplasms. Int J Oncol.

[5] Zaika AI, Kovalev S, Marchenko ND, Moll UM (1999). Overexpression of the wild type p73 gene in breast cancer tissues and cell lines. Cancer Res.

[6] Yokozaki H, Shitara Y, Fujimoto J, Hiyama T, Yasui W, Tahara E (1999). Alterations of p73 preferentially occur in gastric adenocarcinomas with foveolar epithelial phenotype. Int J Cancer.

[7] Yasui W, Yokozaki H, Fujimoto J, Naka K, Kuniyasu H, Tahara E (2000). Genetic and epigenetic alterations in multistep carcinogenesis of the stomach. J Gastroenterol.

[8] Ganini C, Amelio I, Bertolo R, Bove P, Buonomo OC, Candi E, et al. Global mapping of cancers: the cancer genome atlas and beyond. Mol Oncol. 2021. 10.1002/1878-0261.13056 PMC8564642 34245122

[9] Mai M, Yokomizo A, Qian C, Yang P, Tindall DJ, Smith DI (1998). Activation of p73 silent allele in lung cancer. Cancer Res.

[10] He Y, Fan S, Jiang Y, Chen J, Li Z, Zhang H (2000). Study on the transcript expression of p73 gene in human non-small cell lung cancer tissues. Zhongguo Fei Ai Za Zhi.

[11] He Y, Fan S, Jiang Y, Xue Z (2006). Expression of ΔNp73 in human NSCLC and clinical implication. Zhongguo Fei Ai Za Zhi.

[12] Dominguez G, Silva JM, Silva J, Garcia JM, Sanchez A, Navarro A (2001). Wild type p73 overexpression and high-grade malignancy in breast cancer. Breast Cancer Res Treat.

[13] Ahomadegbe JC, Tourpin S, Kaghad M, Zelek L, Vayssade M, Mathieu MC (2000). Loss of heterozygosity, allele silencing and decreased expression of p73 gene in breast cancers: prevalence of alterations in inflammatory breast cancers. Oncogene.

[14] Tomkova K, Belkhiri A, El-Rifai W, Zaika AI (2004). p73 isoforms can induce T-cell factor-dependent transcription in gastrointestinal cells. Cancer Res.

[15] Kamiya M, Nakazato Y (2002). The expression of p73, p21 and MDM2 proteins in gliomas. J Neurooncol.

[16] Wager M, Guilhot J, Blanc JL, Ferrand S, Milin S, Bataille B (2006). Prognostic value of increase in transcript levels of Tp73 DeltaEx2-3 isoforms in low-grade glioma patients. Br J Cancer.

[17] Ugur H, Sayan AE, Ozdamar SO, Kanpolat Y, Ozturk M (2004). Expression of TAP73 and DeltaNP73 in malignant gliomas. Oncol Rep.

[18] Inoue K, Fry EA (2014). Alterations of p63 and p73 in human cancers. Subcell Biochem.

[19] Engelmann D, Meier C, Alla V, Pützer BM (2015). A balancing act: orchestrating amino-truncated and full-length p73 variants as decisive factors in cancer progression. Oncogene.

[20] Tomasini R, Tsuchihara K, Wilhelm M, Fujitani M, Rufini A, Cheung CC (2008). TAp73 knockout shows genomic instability with infertility and tumor suppressor functions. Genes Dev.

[21] Cam M, Charan M, Welker AM, Dravid P, Studebaker AW, Leonard JR (2020). ΔNp73/ETS2 complex drives glioblastoma pathogenesis-targeting downstream mediators by rebastinib prolongs survival in preclinical models of glioblastoma. Neuro Oncol.

[22] Ye H, Guo X. TP73 is a credible biomarker for predicting clinical progression and prognosis in cervical cancer patients. Biosci Rep. 2019;39(8) 10.1042/BSR20190095 PMC6682548 31332036

[23] Tang Z, Li C, Kang B, Gao G, Li C, Zhang Z (2017). GEPIA: a web server for cancer and normal gene expression profiling and interactive analyses. Nucl Acids Res.

[24] Fontemaggi G, Kela I, Amariglio N, Rechavi G, Krishnamurthy J, Strano S (2002). Identification of direct p73 target genes combining DNA microarray and chromatin immunoprecipitation analyses. J Biol Chem.

[25] Urist M, Tanaka T, Poyurovsky MV, Prives C (2004). p73 induction after DNA damage is regulated by checkpoint kinases Chk1 and Chk2. Genes Dev.

[26] Sang M, Ando K, Okoshi R, Koida N, Li Y, Zhu Y (2009). Plk3 inhibits pro-apoptotic activity of p73 through physical interaction and phosphorylation. Genes Cells.

[27] Logotheti S, Michalopoulos I, Sideridou M, Daskalos A, Kossida S, Spandidos DA (2010). Sp1 binds to the external promoter of the p73 gene and induces the expression of TAp73gamma in lung cancer. FEBS J.

[28] Gaiddon C, Lokshin M, Gross I, Levasseur D, Taya Y, Loeffler J-P (2003). Cyclin-dependent kinases phosphorylate p73 at threonine 86 in a cell cycle-dependent manner and negatively regulate p73. J Biol Chem.

[29] Koida N, Ozaki T, Yamamoto H, Ono S, Koda T, Ando K (2008). Inhibitory role of Plk1 in the regulation of p73-dependent apoptosis through physical interaction and phosphorylation. J Biol Chem.

[30] Kostecka A, Sznarkowska A, Meller K, Acedo P, Shi Y, Mohammad Sakil HA, et al. JNK-NQO1 axis drives TAp73-mediated tumor suppression upon oxidative and proteasomal stress. Cell Death Dis. 2014;5:e1484. 10.1038/cddis.2014.408 PMC4649515 25341038

[31] Ren C, Zhang J, Yan W, Zhang Y, Chen X (2016). RNA-binding protein PCBP2 regulates p73 expression and p73-dependent antioxidant defense. J Biol Chem.

[32] Jiang P, Du W, Yang X (2013). A critical role of glucose-6-phosphate dehydrogenase in TAp73-mediated cell proliferation. Cell Cycle.

[33] Li L, Li L, Li W, Chen T, Bin Z, Zhao L (2018). TAp73-induced phosphofructokinase-1 transcription promotes the Warburg effect and enhances cell proliferation. Nat Commun.

[34] Vikhreva P, Petrova V, Gokbulut T, Pestlikis I, Mancini M, Di Daniele N (2017). TAp73 upregulates IL-1β in cancer cells: potential biomarker in lung and breast cancer?. Biochem Biophys Res Commun.

[35] Uramoto H, Sugio K, Oyama T, Nakata S, Ono K, Morita M (2004). Expression of deltaNp73 predicts poor prognosis in lung cancer. Clin Cancer Res.

[36] Wang B, Liu X, Liu H, Guo J, Zhang T, Zhou N, et al. Differential expressions of MDM2 and TAP73 in cancer and cancer-adjacent tissues in patients with non-small-cell lung carcinoma. Pulmonology. 2018. 10.1016/j.rppnen.2017.08.008 29452959

[37] Amelio I, Inoue S, Markert EK, Levine AJ, Knight RA, Mak TW (2015). TAp73 opposes tumor angiogenesis by promoting hypoxia-inducible factor 1α degradation. Proc Natl Acad Sci USA.

[38] Uramoto H, Sugio K, Oyama T, Nakata S, Ono K, Nozoe T (2006). Expression of the p53 family in lung cancer. Anticancer Res.

[39] Wang J, Zheng T, Chen X, Song X, Meng X, Bhatta N (2011). MDM2 antagonist can inhibit tumor growth in hepatocellular carcinoma with different types of p53 in vitro. J Gastroenterol Hepatol.

[40] Yang A, Zhu Z, Kettenbach A, Kapranov P, McKeon F, Gingeras TR, et al. Genome-wide mapping indicates that p73 and p63 co-occupy target sites and have similar dna-binding profiles in vivo. PLoS ONE. 2010;5(7):e11572. 10.1371/journal.pone.0011572 PMC2904373 20644729

[41] Zhang Q, Di C, Yan J, Wang F, Qu T, Wang Y (2019). Inhibition of SF3b1 by pladienolide B evokes cycle arrest, apoptosis induction and p73 splicing in human cervical carcinoma cells. Artif Cells Nanomed Biotechnol.

[42] Oh YK, Lee HJ, Jeong M-H, Rhee M, Mo J-W, Song EH (2008). Role of activating transcription factor 3 on TAp73 stability and apoptosis in paclitaxel-treated cervical cancer cells. Mol Cancer Res.

[43] Wakatsuki M, Ohno T, Iwakawa M, Ishikawa H, Noda S, Ohta T (2008). p73 protein expression correlates with radiation-induced apoptosis in the lack of p53 response to radiation therapy for cervical cancer. Int J Radiat Oncol Biol Phys.

[44] Mega Tiber P, Baloglu L, Ozden S, Ozgen Z, Ozyurt H, Eren M (2014). The association of apoptotic protein expressions sensitive to apoptosis gene, p73 and p53 with the prognosis of cervical carcinoma. Onco Targets Ther.

[45] Schipper H, Alla V, Meier C, Nettelbeck DM, Herchenröder O, Pützer BM (2014). Eradication of metastatic melanoma through cooperative expression of RNA-based HDAC1 inhibitor and p73 by oncolytic adenovirus. Oncotarget.

[46] Steder M, Alla V, Meier C, Spitschak A, Pahnke J, Fürst K (2013). DNp73 exerts function in metastasis initiation by disconnecting the inhibitory role of EPLIN on IGF1R-AKT/STAT3 signaling. Cancer Cell.

[47] Koeppel M, van Heeringen SJ, Kramer D, Smeenk L, Janssen-Megens E, Hartmann M (2011). Crosstalk between c-Jun and TAp73alpha/beta contributes to the apoptosis-survival balance. Nucl Acids Res.

[48] Zhou X, Hao Q, Zhang Q, Liao JM, Ke JW, Liao P (2015). Ribosomal proteins L11 and L5 activate TAp73 by overcoming MDM2 inhibition. Cell Death Differ.

[49] Ohtsuka T, Ryu H, Minamishima YA, Ryo A, Lee SW (2003). Modulation of p53 and p73 levels by cyclin G: implication of a negative feedback regulation. Oncogene.

[50] Ozaki T, Sugimoto H, Nakamura M, Hiraoka K, Yoda H, Sang M (2015). Runt-related transcription factor 2 attenuates the transcriptional activity as well as DNA damage-mediated induction of pro-apoptotic TAp73 to regulate chemosensitivity. FEBS J.

[51] Pediconi N, Guerrieri F, Vossio S, Bruno T, Belloni L, Schinzari V (2009). hSirT1-dependent regulation of the PCAF-E2F1-p73 apoptotic pathway in response to DNA damage. Mol Cell Biol.

[52] Wilhelm MT, Rufini A, Wetzel MK, Tsuchihara K, Inoue S, Tomasini R (2010). Isoform-specific p73 knockout mice reveal a novel role for delta Np73 in the DNA damage response pathway. Genes Dev.

[53] Nakagawa T, Takahashi M, Ozaki T, Watanabe Ki K, Todo S, Mizuguchi H (2002). Autoinhibitory regulation of p73 by Delta Np73 to modulate cell survival and death through a p73-specific target element within the Delta Np73 promoter. Mol Cell Biol.

[54] Amelio I, Markert EK, Rufini A, Antonov AV, Sayan BS, Tucci P (2014). p73 regulates serine biosynthesis in cancer. Oncogene.

[55] Du W, Jiang P, Mancuso A, Stonestrom A, Brewer MD, Minn AJ (2013). TAp73 enhances the pentose phosphate pathway and supports cell proliferation. Nat Cell Biol.

[56] Funato K, Hayashi T, Echizen K, Negishi L, Shimizu N, Koyama-Nasu R, et al. SIRT2-mediated inactivation of p73 is required for glioblastoma tumorigenicity. EMBO Rep. 2018;19(11). 10.15252/embr.201745587 PMC6216266 30213795

[57] Cheng C, Feng S, Jiao J, Huang W, Huang J, Wang L (2018). DLC2 inhibits development of glioma through regulating the expression ratio of TAp73α/TAp73β. Am J Cancer Res.

[58] Casciano I, Mazzocco K, Boni L, Pagnan G, Banelli B, Allemanni G (2002). Expression of DeltaNp73 is a molecular marker for adverse outcome in neuroblastoma patients. Cell Death Differ.

[59] Landré V, Antonov A, Knight R, Melino G (2016). p73 promotes glioblastoma cell invasion by directly activating POSTN (periostin) expression. Oncotarget.

[60] Boominathan L (2010). The guardians of the genome (p53, TA-p73, and TA-p63) are regulators of tumor suppressor miRNAs network. Cancer Metastasis Rev.

[61] Niklison-Chirou MV, Erngren I, Engskog M, Haglöf J, Picard D, Remke M (2017). TAp73 is a marker of glutamine addiction in medulloblastoma. Genes Dev.

[62] Zitterbart K, Zavrelova I, Kadlecova J, Spesna R, Kratochvilova A, Pavelka Z (2007). p73 expression in medulloblastoma: TAp73/DeltaNp73 transcript detection and possible association of p73alpha/DeltaNp73 immunoreactivity with survival. Acta Neuropathol.

[63] Drakos E, Singh RR, Rassidakis GZ, Schlette E, Li J, Claret FX (2011). Activation of the p53 pathway by the MDM2 inhibitor nutlin-3a overcomes BCL2 overexpression in a preclinical model of diffuse large B-cell lymphoma associated with t(14;18)(q32;q21). Leukemia.

[64] Wang Y, Wang X, Flores ER, Yu J, Chang S (2016). Dysfunctional telomeres induce p53-dependent and independent apoptosis to compromise cellular proliferation and inhibit tumor formation. Aging Cell.

[65] Riley MF, You MJ, Multani AS, Lozano G (2016). Mdm2 overexpression and p73 loss exacerbate genomic instability and dampen apoptosis, resulting in B-cell lymphoma. Oncogene.

[66] Nemajerova A, Petrenko O, Trümper L, Palacios G, Moll UM (2010). Loss of p73 promotes dissemination of Myc-induced B cell lymphomas in mice. J Clin Invest.

[67] Feeley KP, Adams CM, Mitra R, Eischen CM (2017). Mdm2 is required for survival and growth of p53-deficient cancer cells. Cancer Res.

[68] Kaida A, Ariumi Y, Ueda Y, Lin JY, Hijikata M, Ikawa S (2000). Functional impairment of p73 and p51, the p53-related proteins, by the human T-cell leukemia virus type 1 Tax oncoprotein. Oncogene.

[69] Chakraborty J, Banerjee S, Ray P, Hossain DMS, Bhattacharyya S, Adhikary A (2010). Gain of cellular adaptation due to prolonged p53 impairment leads to functional switchover from p53 to p73 during DNA damage in acute myeloid leukemia cells. J Biol Chem.

[70] Hanks TS, Gauss KA (2012). Pleomorphic adenoma gene-like 2 regulates expression of the p53 family member, p73, and induces cell cycle block and apoptosis in human promonocytic U937 cells. Apoptosis.

[71] Kawahara M, Hori T, Chonabayashi K, Oka T, Sudol M, Uchiyama T (2008). Kpm/Lats2 is linked to chemosensitivity of leukemic cells through the stabilization of p73. Blood.

[72] Meier M, den Boer ML, Meijerink JPP, Broekhuis MJC, Passier MMCJ, van Wering ER (2006). Differential expression of p73 isoforms in relation to drug resistance in childhood T-lineage acute lymphoblastic leukaemia. Leukemia.

[73] Tebbi A, Guittet O, Cottet M-H, Vesin M-F, Lepoivre M (2011). TAp73 induction by nitric oxide: regulation by checkpoint kinase 1 (CHK1) and protection against apoptosis. J Biol Chem.

[74] de Oliveira RH, Cortez AP, de Ávila RI, da Silva ACG, de Carvalho FS, Menegatti R (2020). Small-molecule MDM2 inhibitor LQFM030-induced apoptosis in p53-null K562 chronic myeloid leukemia cells. Fundam Clin Pharmacol.

[75] Sampath D, Calin GA, Puduvalli VK, Gopisetty G, Taccioli C, Liu C-G (2009). Specific activation of microRNA106b enables the p73 apoptotic response in chronic lymphocytic leukemia by targeting the ubiquitin ligase Itch for degradation. Blood.

[76] Soond SM, Barry SP, Melino G, Knight RA, Latchman DS, Stephanou A (2008). p73-mediated transcriptional activity is negatively regulated by polo-like kinase 1. Cell Cycle.

[77] Tiwary R, Yu W, Sanders BG, Kline K (2011). α-TEA cooperates with chemotherapeutic agents to induce apoptosis of p53 mutant, triple-negative human breast cancer cells via activating p73. Breast Cancer Res.

[78] Domínguez G, García JM, Peña C, Silva J, García V, Martínez L (2006). DeltaTAp73 upregulation correlates with poor prognosis in human tumors: putative in vivo network involving p73 isoforms, p53, and E2F–1. J Clin Oncol.

[79] Strano S, Munarriz E, Rossi M, Cristofanelli B, Shaul Y, Castagnoli L (2000). Physical and functional interaction between p53 mutants and different isoforms of p73. J Biol Chem.

[80] Avraham A, Feldman S, Cho SS, Kol A, Heler L, Riklin-Nahmias E, et al. Breast-specific epigenetic regulation of DeltaNp73 and its role in DNA-damage-response of BRCA1-mutated human mammary epithelial cells. Cancers (Basel). 2020;12(9). 10.3390/cancers12092367 PMC7564633 32825620

[81] Lefkimmiatis K, Caratozzolo MF, Merlo P, D’Erchia AM, Navarro B, Levrero M (2009). p73 and p63 sustain cellular growth by transcriptional activation of cell cycle progression genes. Cancer Res.

[82] Gomez LC, Sottile ML, Guerrero-Gimenez ME, Zoppino FCM, Redondo AL, Gago FE (2018). TP73 DNA methylation and upregulation of ΔNp73 are associated with an adverse prognosis in breast cancer. J Clin Pathol.

[83] Lemos A, Gomes AS, Loureiro JB, Brandão P, Palmeira A, Pinto MMM, et al. Synthesis, biological evaluation, and in silico studies of novel aminated xanthones as potential p53-activating agents. Molecules. 2019;24(10) 10.3390/molecules24101975 PMC6571851 31121972

[84] Soldevilla B, Díaz R, Silva J, Campos-Martín Y, Muñoz C, García V (2011). Prognostic impact of ΔTAp73 isoform levels and their target genes in colon cancer patients. Clin Cancer Res.

[85] Jung MS, Yun J, Chae HD, Kim JM, Kim SC, Choi TS (2001). p53 and its homologues, p63 and p73, induce a replicative senescence through inactivation of NF-Y transcription factor. Oncogene.

[86] Wojdyla L, Stone AL, Sethakorn N, Uppada SB, Devito JT, Bissonnette M (2014). T-oligo as an anticancer agent in colorectal cancer. Biochem Biophys Res Commun.

[87] Rufini A, Niklison-Chirou MV, Inoue S, Tomasini R, Harris IS, Marino A (2012). TAp73 depletion accelerates aging through metabolic dysregulation. Genes Dev.

[88] Gong H, Zhang Y, Jiang K, Ye S, Chen S, Zhang Q (2018). p73 coordinates with Δ133p53 to promote DNA double-strand break repair. Cell Death Differ.

[89] Domínguez G, Peña C, Silva J, García JM, García V, Rodríguez R (2006). The presence of an intronic deletion in p73 and high levels of ZEB1 alter the TAp73/DeltaTAp73 ratio in colorectal carcinomas. J Pathol.

[90] Deng X, Sheng J, Liu H, Wang N, Dai C, Wang Z (2019). Cinobufagin promotes cell cycle arrest and apoptosis to block human esophageal squamous cell carcinoma cells growth via the p73 signalling pathway. Biol Pharm Bull.

[91] Zaika E, Bhardwaj V, Wei J, Washington MK, Souza R, El-Rifai W, et al. Proinflammatory cytokines and bile acids upregulate ΔNp73 protein, an inhibitor of p53 and p73 tumor suppressors. PLoS ONE. 2013;8(5):e64306. 10.1371/journal.pone.0064306 PMC3661465 23717592

[92] Bhardwaj V, Horvat A, Korolkova O, Washington MK, El-Rifai W, Dikalov SI (2016). Prevention of DNA damage in Barrett’s esophageal cells exposed to acidic bile salts. Carcinogenesis.

[93] Zaika E, Wei J, Yin D, Andl C, Moll U, El-Rifai W (2011). p73 protein regulates DNA damage repair. FASEB J.

[94] Vilgelm AE, Hong SM, Washington MK, Wei J, Chen H, El-Rifai W (2010). Characterization of ΔNp73 expression and regulation in gastric and esophageal tumors. Oncogene.

[95] Malaguarnera R, Vella V, Pandini G, Sanfilippo M, Pezzino V, Vigneri R (2008). TAp73 alpha increases p53 tumor suppressor activity in thyroid cancer cells via the inhibition of Mdm2-mediated degradation. Mol Cancer Res.

[96] Concin N, Hofstetter G, Berger A, Gehmacher A, Reimer D, Watrowski R (2005). Clinical relevance of dominant-negative p73 isoforms for responsiveness to chemotherapy and survival in ovarian cancer: evidence for a crucial p53–p73 cross-talk in vivo. Clin Cancer Res.

[97] Vikhanskaya F, D’Incalci M, Broggini M (2000). p73 competes with p53 and attenuates its response in a human ovarian cancer cell line. Nucl Acids Res.

[98] Hofstetter G, Berger A, Chamson M, Müller-Holzner E, Reimer D, Ulmer H (2011). Clinical relevance of TAp73 and ΔNp73 protein expression in ovarian cancer: a series of 83 cases and review of the literature. Int J Gynecol Pathol.

[99] Chen J, Li D, Killary AM, Sen S, Amos CI, Evans DB (2009). Polymorphisms of p16, p27, p73, and MDM2 modulate response and survival of pancreatic cancer patients treated with preoperative chemoradiation. Ann Surg Oncol.

[100] Ito Y, Takeda T, Wakasa K, Tsujimoto M, Sakon M, Matsuura N (2001). Expression of p73 and p63 proteins in pancreatic adenocarcinoma: p73 overexpression is inversely correlated with biological aggressiveness. Int J Mol Med.

[101] Ozaki T, Hosoda M, Miyazaki K, Hayashi S, Watanabe K-I, Nakagawa T (2005). Functional implication of p73 protein stability in neuronal cell survival and death. Cancer Lett.

[102] Gomes S, Raimundo L, Soares J, Loureiro JB, Leão M, Ramos H (2019). New inhibitor of the TAp73 interaction with MDM2 and mutant p53 with promising antitumor activity against neuroblastoma. Cancer Lett.

[103] Rossi M, Sayan AE, Terrinoni A, Melino G, Knight RA (2004). Mechanism of induction of apoptosis by p73 and its relevance to neuroblastoma biology. Ann N Y Acad Sci.

[104] Zekri A, Ghaffari SH, Yaghmaie M, Estiar MA, Alimoghaddam K, Modarressi MH (2016). Inhibitor of aurora kinase B induces differentially cell death and polyploidy via DNA damage response pathways in neurological malignancy: shedding new light on the challenge of resistance to AZD1152-HQPA. Mol Neurobiol.

[105] Sinha N, Panda PK, Naik PP, Das DN, Mukhopadhyay S, Maiti TK (2017). Abrus agglutinin promotes irreparable DNA damage by triggering ROS generation followed by ATM-p73 mediated apoptosis in oral squamous cell carcinoma. Mol Carcinog.

[106] Lu H, Yang X, Duggal P, Allen CT, Yan B, Cohen J (2011). TNF-α promotes c-REL/ΔNp63α interaction and TAp73 dissociation from key genes that mediate growth arrest and apoptosis in head and neck cancer. Cancer Res.

[107] Li J, Jiang X, Zhou X (2005). Expression and prognosis significance of p73 and PCNA in laryngeal squamous cell carcinoma. Lin Chuang Er Bi Yan Hou Ke Za Zhi.

[108] Choi H-R, Batsakis JG, Zhan F, Sturgis E, Luna MA, El-Naggar AK (2002). Differential expression of p53 gene family members p63 and p73 in head and neck squamous tumorigenesis. Hum Pathol.

[109] Velletri T, Huang Y, Wang Y, Li Q, Hu M, Xie N (2021). Loss of p53 in mesenchymal stem cells promotes alteration of bone remodeling through negative regulation of osteoprotegerin. Cell Death Differ.

[110] Radine C, Peters D, Reese A, Neuwahl J, Budach W, Jänicke RU (2020). The RNA-binding protein RBM47 is a novel regulator of cell fate decisions by transcriptionally controlling the p53–p21-axis. Cell Death Differ.

[111] Hanahan D, Weinberg RA (2011). Hallmarks of cancer: the next generation. Cell.

[112] Marin MC, Marques MM. Novel role of p73 as a regulator of developmental angiogenesis: implication for cancer therapy. Mol Cell Oncol. 2016;3(1):e1019973. 10.1080/23723556.2015.1019973 PMC4845169 27308533

[113] Sabapathy K (2015). p73: a positive or negative regulator of angiogenesis, or both?. Mol Cell Biol.

[114] Napoli M, Flores ER (2017). The p53 family orchestrates the regulation of metabolism: physiological regulation and implications for cancer therapy. Br J Cancer.

[115] Itahana Y, Itahana K. Emerging roles of p53 family members in glucose metabolism. Int J Mol Sci. 2018;19(3). 10.3390/ijms19030776 PMC5877637 29518025

[116] Nemajerova A, Amelio I, Gebel J, Dötsch V, Melino G, Moll UM (2018). Non-oncogenic roles of TAp73: from multiciliogenesis to metabolism. Cell Death Differ.

[117] Belyi VA, Ak P, Markert E, Wang H, Hu W, Puzio-Kuter A, et al. The origins and evolution of the p53 family of genes. Cold Spring Harb Perspect Biol. 2010;2(6):a001198. 10.1101/cshperspect.a001198 PMC2869528 20516129

[118] Murray-Zmijewski F, Lane DP, Bourdon JC (2006). p53/p63/p73 isoforms: an orchestra of isoforms to harmonise cell differentiation and response to stress. Cell Death Differ.

[119] Jost CA, Marin MC, Kaelin WG (1997). p73 is a simian [correction of human] p53-related protein that can induce apoptosis. Nature.

[120] Kaghad M, Bonnet H, Yang A, Creancier L, Biscan JC, Valent A (1997). Monoallelically expressed gene related to p53 at 1p36, a region frequently deleted in neuroblastoma and other human cancers. Cell.

[121] Zeng X, Li X, Miller A, Yuan Z, Yuan W, Kwok RP (2000). The N-terminal domain of p73 interacts with the CH1 domain of p300/CREB binding protein and mediates transcriptional activation and apoptosis. Mol Cell Biol.

[122] Yang A, Walker N, Bronson R, Kaghad M, Oosterwegel M, Bonnin J (2000). p73-deficient mice have neurological, pheromonal and inflammatory defects but lack spontaneous tumours. Nature.

[123] Stiewe T, Theseling CC, Pützer BM (2002). Transactivation-deficient Delta TA-p73 inhibits p53 by direct competition for DNA binding: implications for tumorigenesis. J Biol Chem.

[124] Pozniak CD, Radinovic S, Yang A, McKeon F, Kaplan DR, Miller FD (2000). An anti-apoptotic role for the p53 family member, p73, during developmental neuron death. Science.

[125] Concin N, Becker K, Slade N, Erster S, Mueller-Holzner E, Ulmer H, et al. Transdominant DeltaTAp73 isoforms are frequently up-regulated in ovarian cancer. Evidence for their role as epigenetic p53 inhibitors in vivo. Cancer Res. 2004;64(7):2449–60. 10.1158/0008-5472.can-03-1060 15059898

[126] Nakagawa T, Takahashi M, Ozaki T, Watanabe K, Hayashi S, Hosoda M (2003). Negative autoregulation of p73 and p53 by DeltaNp73 in regulating differentiation and survival of human neuroblastoma cells. Cancer Lett.

[127] Liu G, Nozell S, Xiao H, Chen X (2004). DeltaNp73beta is active in transactivation and growth suppression. Mol Cell Biol.

[128] Beeler JS, Marshall CB, Gonzalez-Ericsson PI, Shaver TM, Santos Guasch GL, Lea ST, et al. p73 regulates epidermal wound healing and induced keratinocyte programming. PLoS ONE. 2019;14(6):e0218458. 10.1371/journal.pone.0218458 PMC6583996 31216312

[129] Sánchez-Carrera D, García-Puga M, Yáñez L, Romón Í, Pipaón C. ∆Np73 is capable of inducing apoptosis by co-ordinately activating several BH3-only proteins. Biosci Rep. 2015;35(3). 10.1042/BSR20150039 PMC4613676 26182360

[130] Nyman U, Vlachos P, Cascante A, Hermanson O, Zhivotovsky B, Joseph B (2009). Protein kinase C-dependent phosphorylation regulates the cell cycle-inhibitory function of the p73 carboxy terminus transactivation domain. Mol Cell Biol.

[131] Ho WC, Fitzgerald MX, Marmorstein R (2006). Structure of the p53 core domain dimer bound to DNA. J Biol Chem.

[132] Joerger AC, Wilcken R, Andreeva A (2014). Tracing the evolution of the p53 tetramerization domain. Structure.

[133] Levrero M, De Laurenzi V, Costanzo A, Gong J, Wang JY, Melino G (2000). The p53/p63/p73 family of transcription factors: overlapping and distinct functions. J Cell Sci.

[134] Gebel J, Luh LM, Coutandin D, Osterburg C, Löhr F, Schäfer B (2016). Mechanism of TAp73 inhibition by ΔNp63 and structural basis of p63/p73 hetero-tetramerization. Cell Death Differ.

[135] Gatti V, Fierro C, Annicchiarico-Petruzzelli M, Melino G, Peschiaroli A (2019). ΔNp63 in squamous cell carcinoma: defining the oncogenic routes affecting epigenetic landscape and tumour microenvironment. Mol Oncol.

[136] Liu G, Chen X (2005). The C-terminal sterile alpha motif and the extreme C terminus regulate the transcriptional activity of the alpha isoform of p73. J Biol Chem.

[137] Vikhreva P, Melino G, Amelio I (2018). p73 alternative splicing: exploring a biological role for the C-terminal isoforms. J Mol Biol.

[138] Vikhanskaya F, Toh WH, Dulloo I, Wu Q, Boominathan L, Ng HH (2007). p73 supports cellular growth through c-Jun-dependent AP-1 transactivation. Nat Cell Biol.

[139] Subramanian D, Bunjobpol W, Sabapathy K (2015). Interplay between TAp73 protein and selected activator protein-1 (AP-1) family members promotes AP-1 target gene activation and cellular growth. J Biol Chem.

[140] Si H, Lu H, Yang X, Mattox A, Jang M, Bian Y (2016). TNF-α modulates genome-wide redistribution of ΔNp63α/TAp73 and NF-κB cREL interactive binding on TP53 and AP-1 motifs to promote an oncogenic gene program in squamous cancer. Oncogene.

[141] Watson IR, Blanch A, Lin DCC, Ohh M, Irwin MS (2006). Mdm2-mediated NEDD8 modification of TAp73 regulates its transactivation function. J Biol Chem.

[142] Nieto A, Hara MR, Quereda V, Grant W, Saunders V, Xiao K (2020). βarrestin-1 regulates DNA repair by acting as an E3-ubiquitin ligase adaptor for 53BP1. Cell Death Differ.

[143] Li X, Guo M, Cai L, Du T, Liu Y, Ding H-F (2020). Competitive ubiquitination activates the tumor suppressor p53. Cell Death Differ.

[144] Long JS, Schoonen PM, Graczyk D, O’Prey J, Ryan KM (2015). p73 engages A2B receptor signalling to prime cancer cells to chemotherapy-induced death. Oncogene.

[145] Ozaki T, Nakagawara A (2005). p73, a sophisticated p53 family member in the cancer world. Cancer Sci.

[146] Vossio S, Palescandolo E, Pediconi N, Moretti F, Balsano C, Levrero M (2002). DN-p73 is activated after DNA damage in a p53-dependent manner to regulate p53-induced cell cycle arrest. Oncogene.

[147] Grob TJ, Novak U, Maisse C, Barcaroli D, Lüthi AU, Pirnia F (2001). Human delta Np73 regulates a dominant negative feedback loop for TAp73 and p53. Cell Death Differ.

[148] Kartasheva NN, Contente A, Lenz-Stöppler C, Roth J, Dobbelstein M (2002). p53 induces the expression of its antagonist p73 Delta N, establishing an autoregulatory feedback loop. Oncogene.

[149] Oswald C, Stiewe T (2008). In good times and bad: p73 in cancer. Cell Cycle.

[150] Meier C, Hardtstock P, Joost S, Alla V, Pützer BM (2016). p73 and IGF1R regulate emergence of aggressive cancer stem-like features via miR-885-5p control. Cancer Res.

[151] Logotheti S, Pavlopoulou A, Galtsidis S, Vojtesek B, Zoumpourlis V (2013). Functions, divergence and clinical value of TAp73 isoforms in cancer. Cancer Metastasis Rev.

[152] Li W, Zhang X, Xi X, Li Y, Quan H, Liu S (2020). PLK2 modulation of enriched TAp73 affects osteogenic differentiation and prognosis in human osteosarcoma. Cancer Med.

[153] Fürst K, Steder M, Logotheti S, Angerilli A, Spitschak A, Marquardt S (2019). DNp73-induced degradation of tyrosinase links depigmentation with EMT-driven melanoma progression. Cancer Lett.

[154] Di C, Yang L, Zhang H, Ma X, Zhang X, Sun C (2013). Mechanisms, function and clinical applications of DNp73. Cell Cycle.

[155] Conforti F, Yang AL, Agostini M, Rufini A, Tucci P, Nicklison-Chirou MV (2012). Relative expression of TAp73 and ΔNp73 isoforms. Aging (Albany NY).

[156] Kravchenko JE, Ilyinskaya GV, Komarov PG, Agapova LS, Kochetkov DV, Strom E (2008). Small-molecule RETRA suppresses mutant p53-bearing cancer cells through a p73-dependent salvage pathway. Proc Natl Acad Sci USA.

[157] Alsafadi S, Tourpin S, André F, Vassal G, Ahomadegbe J-C (2009). P53 family: at the crossroads in cancer therapy. Curr Med Chem.

[158] Sonnemann J, Grauel D, Blümel L, Hentschel J, Marx C, Blumrich A (2015). RETRA exerts anticancer activity in Ewing’s sarcoma cells independent of their TP53 status. Eur J Cancer.

[159] Fernandez-Alonso R, Martin-Lopez M, Gonzalez-Cano L, Garcia S, Castrillo F, Diez-Prieto I (2015). p73 is required for endothelial cell differentiation, migration and the formation of vascular networks regulating VEGF and TGFβ signaling. Cell Death Differ.

[160] Niklison-Chirou MV, Steinert JR, Agostini M, Knight RA, Dinsdale D, Cattaneo A (2013). TAp73 knockout mice show morphological and functional nervous system defects associated with loss of p75 neurotrophin receptor. Proc Natl Acad Sci USA.

[161] Gong JG, Costanzo A, Yang HQ, Melino G, Kaelin WG, Levrero M (1999). The tyrosine kinase c-Abl regulates p73 in apoptotic response to cisplatin-induced DNA damage. Nature.

[162] Wang X, Zeng L, Wang J, Chau JFL, Lai KP, Jia D (2011). A positive role for c-Abl in Atm and Atr activation in DNA damage response. Cell Death Differ.

[163] Yuan ZM, Shioya H, Ishiko T, Sun X, Gu J, Huang YY (1999). p73 is regulated by tyrosine kinase c-Abl in the apoptotic response to DNA damage. Nature.

[164] Reuven N, Shaul Y. The c-Abl/YAP/p73 apoptotic module and the HIPPO pathway. In: Oren M, Aylon Y, editors. The hippo signaling pathway and cancer. Springer, New York; 2013. p. 173–95.

[165] Ibrahim N, He L, Leong C-O, Xing D, Karlan BY, Swisher EM (2010). BRCA1-associated epigenetic regulation of p73 mediates an effector pathway for chemosensitivity in ovarian carcinoma. Cancer Res.

[166] Hastak K, Alli E, Ford JM (2010). Synergistic chemosensitivity of triple-negative breast cancer cell lines to poly(ADP-Ribose) polymerase inhibition, gemcitabine, and cisplatin. Cancer Res.

[167] Rishi V, Bhattacharya P, Chatterjee R, Rozenberg J, Zhao J, Glass K (2010). CpG methylation of half-CRE sequences creates C/EBPalpha binding sites that activate some tissue-specific genes. Proc Natl Acad Sci USA.

[168] Rozenberg JM, Bhattacharya P, Chatterjee R, Glass K, Vinson C. Combinatorial recruitment of CREB, C/EBPβ and c-Jun determines activation of promoters upon keratinocyte differentiation. PLoS ONE. 2013;8(11):e78179. 10.1371/journal.pone.0078179 PMC3820678 24244291

[169] Rozenberg JM, Taylor JM, Mack CP (2018). RBPJ binds to consensus and methylated cis elements within phased nucleosomes and controls gene expression in human aortic smooth muscle cells in cooperation with SRF. Nucl Acids Res.

[170] Banin S, Moyal L, Shieh S, Taya Y, Anderson CW, Chessa L (1998). Enhanced phosphorylation of p53 by ATM in response to DNA damage. Science.

[171] Canman CE, Lim DS, Cimprich KA, Taya Y, Tamai K, Sakaguchi K (1998). Activation of the ATM kinase by ionizing radiation and phosphorylation of p53. Science.

[172] Zhang Y-X, Pan W-Y, Chen J (2019). p53 and its isoforms in DNA double-stranded break repair. J Zhejiang Univ Sci B.

[173] Lin Y-L, Sengupta S, Gurdziel K, Bell GW, Jacks T, Flores ER. p63 and p73 transcriptionally regulate genes involved in DNA repair. PLoS Genet. 2009;5(10):e1000680. 10.1371/journal.pgen.1000680 PMC2752189 19816568

[174] Wakasugi T, Izumi H, Uchiumi T, Suzuki H, Arao T, Nishio K (2007). ZNF143 interacts with p73 and is involved in cisplatin resistance through the transcriptional regulation of DNA repair genes. Oncogene.

[175] Gong L, Pan X, Abali GK, Little JB, Yuan Z-M (2020). Functional interplay between p53 and Δ133p53 in adaptive stress response. Cell Death Differ.

[176] Li LS, Morales JC, Hwang A, Wagner MW, Boothman DA (2008). DNA mismatch repair-dependent activation of c-Abl/p73alpha/GADD45alpha-mediated apoptosis. J Biol Chem.

[177] Jackson JG, Pereira-Smith OM (2006). p53 is preferentially recruited to the promoters of growth arrest genes p21 and GADD45 during replicative senescence of normal human fibroblasts. Cancer Res.

[178] Engeland K (2018). Cell cycle arrest through indirect transcriptional repression by p53: I have a DREAM. Cell Death Differ.

[179] Vanzo R, Bartkova J, Merchut-Maya JM, Hall A, Bouchal J, Dyrskjøt L (2020). Autophagy role(s) in response to oncogenes and DNA replication stress. Cell Death Differ.

[180] Li L, Yang G, Ren C, Tanimoto R, Hirayama T, Wang J (2013). Glioma pathogenesis-related protein 1 induces prostate cancer cell death through Hsc70-mediated suppression of AURKA and TPX2. Mol Oncol.

[181] Liu H, Weng W, Guo R, Zhou J, Xue J, Zhong S (2020). Olig2 SUMOylation protects against genotoxic damage response by antagonizing p53 gene targeting. Cell Death Differ.

[182] Irwin MS, Kondo K, Marin MC, Cheng LS, Hahn WC, Kaelin WG (2003). Chemosensitivity linked to p73 function. Cancer Cell.

[183] Flores ER, Tsai KY, Crowley D, Sengupta S, Yang A, McKeon F (2002). p63 and p73 are required for p53-dependent apoptosis in response to DNA damage. Nature.

[184] Tozluoğlu M, Karaca E, Haliloglu T, Nussinov R (2008). Cataloging and organizing p73 interactions in cell cycle arrest and apoptosis. Nucl Acids Res.

[185] Gonzalez S, Prives C, Cordon-Cardo C (2003). p73alpha regulation by Chk1 in response to DNA damage. Mol Cell Biol.

[186] Merlo P, Fulco M, Costanzo A, Mangiacasale R, Strano S, Blandino G (2005). A role of p73 in mitotic exit. J Biol Chem.

[187] Vayssade M, Haddada H, Faridoni-Laurens L, Tourpin S, Valent A, Bénard J (2005). P73 functionally replaces p53 in Adriamycin-treated, p53-deficient breast cancer cells. Int J Cancer.

[188] Fulco M, Costanzo A, Merlo P, Mangiacasale R, Strano S, Blandino G (2003). p73 is regulated by phosphorylation at the G2/M transition. J Biol Chem.

[189] Seelan RS, Irwin M, van der Stoop P, Qian C, Kaelin WG, Liu W (2002). The human p73 promoter: characterization and identification of functional E2F binding sites. Neoplasia.

[190] Wang JY, Ki SW (2001). Choosing between growth arrest and apoptosis through the retinoblastoma tumour suppressor protein, Abl and p73. Biochem Soc Trans.

[191] Finzer P, Krueger A, Stöhr M, Brenner D, Soto U, Kuntzen C (2004). HDAC inhibitors trigger apoptosis in HPV-positive cells by inducing the E2F–p73 pathway. Oncogene.

[192] Innocente SA, Lee JM (2005). p73 is a p53-independent, Sp1-dependent repressor of cyclin B1 transcription. Biochem Biophys Res Commun.

[193] Lee CW, La Thangue NB (1999). Promoter specificity and stability control of the p53-related protein p73. Oncogene.

[194] Paruthiyil S, Cvoro A, Tagliaferri M, Cohen I, Shtivelman E, Leitman DC (2011). Estrogen receptor β causes a G2 cell cycle arrest by inhibiting CDK1 activity through the regulation of cyclin B1, GADD45A, and BTG2. Breast Cancer Res Treat.

[195] Wang XW, Zhan Q, Coursen JD, Khan MA, Kontny HU, Yu L (1999). GADD45 induction of a G2/M cell cycle checkpoint. Proc Natl Acad Sci USA.

[196] Wang XQ, Ongkeko WM, Lau AW, Leung KM, Poon RY (2001). A possible role of p73 on the modulation of p53 level through MDM2. Cancer Res.

[197] Vernole P, Neale MH, Barcaroli D, Munarriz E, Knight RA, Tomasini R (2009). TAp73alpha binds the kinetochore proteins Bub1 and Bub3 resulting in polyploidy. Cell Cycle.

[198] Tomasini R, Tsuchihara K, Tsuda C, Lau SK, Wilhelm M, Rufini A (2009). TAp73 regulates the spindle assembly checkpoint by modulating BubR1 activity. Proc Natl Acad Sci USA.

[199] Marrazzo E, Marchini S, Tavecchio M, Alberio T, Previdi S, Erba E (2009). The expression of the DeltaNp73beta isoform of p73 leads to tetraploidy. Eur J Cancer.

[200] Mikulenkova E, Neradil J, Zitterbart K, Sterba J, Veselska R (2015). Overexpression of the ∆Np73 isoform is associated with centrosome amplification in brain tumor cell lines. Tumour Biol.

[201] Katayama H, Wang J, Treekitkarnmongkol W, Kawai H, Sasai K, Zhang H (2012). Aurora kinase-A inactivates DNA damage-induced apoptosis and spindle assembly checkpoint response functions of p73. Cancer Cell.

[202] Sasai K, Treekitkarnmongkol W, Kai K, Katayama H, Sen S (2016). Functional significance of aurora kinases-p53 protein family interactions in cancer. Front Oncol.

[203] Zhang M, Zhang J, Yan W, Chen X (2016). p73 expression is regulated by ribosomal protein RPL26 through mRNA translation and protein stability. Oncotarget.

[204] Yan W, Zhang J, Zhang Y, Jung Y-S, Chen X (2012). p73 expression is regulated by RNPC1, a target of the p53 family, via mRNA stability. Mol Cell Biol.

[205] Salama M, Benitez-Riquelme D, Elabd S, Munoz L, Zhang P, Glanemann M (2019). Fam83F induces p53 stabilisation and promotes its activity. Cell Death Differ.

[206] Zhu T, Tsuji T, Chen C (2010). Roles of PKC isoforms in the induction of apoptosis elicited by aberrant Ras. Oncogene.

[207] Sayan BS, Yang AL, Conforti F, Tucci P, Piro MC, Browne GJ (2010). Differential control of TAp73 and DeltaNp73 protein stability by the ring finger ubiquitin ligase PIR2. Proc Natl Acad Sci USA.

[208] Sun G, Jin S, Baskaran R (2009). MMR/c-Abl-dependent activation of ING2/p73alpha signaling regulates the cell death response to N-methyl-N’-nitro-N-nitrosoguanidine. Exp Cell Res.

[209] Jones EV, Dickman MJ, Whitmarsh AJ (2007). Regulation of p73-mediated apoptosis by c-Jun N-terminal kinase. Biochem J.

[210] Bernassola F, Salomoni P, Oberst A, Di Como CJ, Pagano M, Melino G (2004). Ubiquitin-dependent degradation of p73 is inhibited by PML. J Exp Med.

[211] Shimodaira H, Yoshioka-Yamashita A, Kolodner RD, Wang JYJ (2003). Interaction of mismatch repair protein PMS2 and the p53-related transcription factor p73 in apoptosis response to cisplatin. Proc Natl Acad Sci USA.

[212] Kubo N, Okoshi R, Nakashima K, Shimozato O, Nakagawara A, Ozaki T (2010). MDM2 promotes the proteasomal degradation of p73 through the interaction with Itch in HeLa cells. Biochem Biophys Res Commun.

[213] Wu H, Zeinab RA, Flores ER, Leng RP (2011). Pirh2, a ubiquitin E3 ligase, inhibits p73 transcriptional activity by promoting its ubiquitination. Mol Cancer Res.

[214] Peschiaroli A, Scialpi F, Bernassola F, Pagano M, Melino G (2009). The F-box protein FBXO45 promotes the proteasome-dependent degradation of p73. Oncogene.

[215] Chaudhary N, Maddika S (2014). WWP2-WWP1 ubiquitin ligase complex coordinated by PPM1G maintains the balance between cellular p73 and ΔNp73 levels. Mol Cell Biol.

[216] Munarriz E, Barcaroli D, Stephanou A, Townsend PA, Maisse C, Terrinoni A (2004). PIAS-1 is a checkpoint regulator which affects exit from G1 and G2 by sumoylation of p73. Mol Cell Biol.

[217] Sznarkowska A, Kostecka A, Kawiak A, Acedo P, Lion M, Inga A (2018). Reactivation of TAp73 tumor suppressor by protoporphyrin IX, a metabolite of aminolevulinic acid, induces apoptosis in TP53-deficient cancer cells. Cell Div.

[218] Miyazaki K, Ozaki T, Kato C, Hanamoto T, Fujita T, Irino S (2003). A novel HECT-type E3 ubiquitin ligase, NEDL2, stabilizes p73 and enhances its transcriptional activity. Biochem Biophys Res Commun.

[219] Lu L, Hu S, Wei R, Qiu X, Lu K, Fu Y (2013). The HECT type ubiquitin ligase NEDL2 is degraded by anaphase-promoting complex/cyclosome (APC/C)-Cdh1, and its tight regulation maintains the metaphase to anaphase transition. J Biol Chem.

[220] Ganapathy S, Peng B, Shen L, Yu T, Lafontant J, Li P (2017). Suppression of PKC causes oncogenic stress for triggering apoptosis in cancer cells. Oncotarget.

[221] Satija YK, Das S (2016). Tyr99 phosphorylation determines the regulatory milieu of tumor suppressor p73. Oncogene.

[222] Strano S, Monti O, Pediconi N, Baccarini A, Fontemaggi G, Lapi E (2005). The transcriptional coactivator Yes-associated protein drives p73 gene-target specificity in response to DNA Damage. Mol Cell.

[223] Raj N, Bam R (2019). Reciprocal crosstalk between yap1/hippo pathway and the p53 family proteins: mechanisms and outcomes in cancer. Front Cell Dev Biol.

[224] Mantovani F, Piazza S, Gostissa M, Strano S, Zacchi P, Mantovani R (2004). Pin1 links the activities of c-Abl and p300 in regulating p73 function. Mol Cell.

[225] Dai JM, Wang ZY, Sun DC, Lin RX, Wang SQ (2007). SIRT1 interacts with p73 and suppresses p73-dependent transcriptional activity. J Cell Physiol.

[226] Accardi R, Scalise M, Gheit T, Hussain I, Yue J, Carreira C (2011). IkappaB kinase beta promotes cell survival by antagonizing p53 functions through DeltaNp73alpha phosphorylation and stabilization. Mol Cell Biol.

[227] Otkur W, Wang F, Liu W, Hayashi T, Tashiro S-I, Onodera S (2018). Persistent IKKα phosphorylation induced apoptosis in UVB and Poly I: C co-treated HaCaT cells plausibly through pro-apoptotic p73 and abrogation of IκBα. Mol Immunol.

[228] Chen D, Ming L, Zou F, Peng Y, Van Houten B, Yu J (2014). TAp73 promotes cell survival upon genotoxic stress by inhibiting p53 activity. Oncotarget.

[229] Tracz-Gaszewska Z, Klimczak M, Biecek P, Herok M, Kosinski M, Olszewski MB (2017). Molecular chaperones in the acquisition of cancer cell chemoresistance with mutated TP53 and MDM2 up-regulation. Oncotarget.

[230] Wolf ER, McAtarsney CP, Bredhold KE, Kline AM, Mayo LD. Mutant and wild-type p53 form complexes with p73 upon phosphorylation by the kinase JNK. Sci Signal. 2018;11(524). 10.1126/scisignal.aao4170 PMC6671681 29615516

[231] Tutaeva VV, Bobin AN, Ovsiannikova MR, Bulgakova MV, Kuchma YM, Kryukov EV, et al. Disseminated form of the Kaposi sarcoma in HIV-negative patient associated with Hodgkin’s lymphoma. Oxf Med Case Rep. 2020;2020(9):omaa069. 10.1093/omcr/omaa069 PMC7507874 32995025

[232] Yi SA, Lee DH, Kim GW, Ryu H-W, Park JW, Lee J (2020). HPV-mediated nuclear export of HP1γ drives cervical tumorigenesis by downregulation of p53. Cell Death Differ.

[233] Chan C, Thurnherr T, Wang J, Gallart-Palau X, Sze SK, Rozen S (2016). Global re-wiring of p53 transcription regulation by the hepatitis B virus X protein. Mol Oncol.

[234] Deb D, Lanyi A, Scian M, Keiger J, Brown DR, Le Roith D (2001). Differential modulation of cellular and viral promoters by p73 and p53. Int J Oncol.

[235] Lemasson I, Nyborg JK (2001). Human T-cell leukemia virus type I tax repression of p73beta is mediated through competition for the C/H1 domain of CBP. J Biol Chem.

[236] Uramoto H, Wetterskog D, Hackzell A, Matsumoto Y, Funa K (2004). p73 competes with co-activators and recruits histone deacetylase to NF-Y in the repression of PDGF beta-receptor. J Cell Sci.

[237] Sudhakar C, Jain N, Swarup G (2008). Sp1-like sequences mediate human caspase-3 promoter activation by p73 and cisplatin. FEBS J.

[238] Grande L, Bretones G, Rosa-Garrido M, Garrido-Martin EM, Hernandez T, Fraile S (2012). Transcription factors Sp1 and p73 control the expression of the proapoptotic protein NOXA in the response of testicular embryonal carcinoma cells to cisplatin. J Biol Chem.

[239] Talos F, Nemajerova A, Flores ER, Petrenko O, Moll UM (2007). p73 suppresses polyploidy and aneuploidy in the absence of functional p53. Mol Cell.

[240] Espinoza JA, Zisi A, Kanellis DC, Carreras-Puigvert J, Henriksson M, Hühn D (2020). The antimalarial drug amodiaquine stabilizes p53 through ribosome biogenesis stress, independently of its autophagy-inhibitory activity. Cell Death Differ.

[241] Wang JY (2000). Regulation of cell death by the Abl tyrosine kinase. Oncogene.

[242] Chen C, Whitney IP, Banerjee A, Sacristan C, Sekhri P, Kern DM (2019). Ectopic activation of the spindle assembly checkpoint signaling cascade reveals its biochemical design. Curr Biol.

[243] Dick AE, Gerlich DW (2013). Kinetic framework of spindle assembly checkpoint signalling. Nat Cell Biol.

[244] Musacchio A (2015). The molecular biology of spindle assembly checkpoint signaling dynamics. Curr Biol.

[245] Lischetti T, Nilsson J. Regulation of mitotic progression by the spindle assembly checkpoint. Mol Cell Oncol. 2015;2(1):e970484. 10.4161/23723548.2014.970484 PMC4905242 27308407

[246] Frank T, Tuppi M, Hugle M, Dötsch V, van Wijk SJL, Fulda S (2019). Cell cycle arrest in mitosis promotes interferon-induced necroptosis. Cell Death Differ.

[247] Chen J, Li P, Song L, Bai L, Huen MSY, Liu Y (2020). 53BP1 loss rescues embryonic lethality but not genomic instability of BRCA1 total knockout mice. Cell Death Differ.

[248] Toh WH, Nam SY, Sabapathy K (2010). An essential role for p73 in regulating mitotic cell death. Cell Death Differ.

[249] Saadatzadeh MR, Elmi AN, Pandya PH, Bijangi-Vishehsaraei K, Ding J, Stamatkin CW, et al. The role of MDM2 in promoting genome stability versus instability. Int J Mol Sci. 2017;18(10). 10.3390/ijms18102216 PMC5666895 29065514

[250] Wei MC, Zong WX, Cheng EH, Lindsten T, Panoutsakopoulou V, Ross AJ (2001). Proapoptotic BAX and BAK: a requisite gateway to mitochondrial dysfunction and death. Science.

[251] Julien O, Wells JA (2017). Caspases and their substrates. Cell Death Differ.

[252] Li LY, Luo X, Wang X (2001). Endonuclease G is an apoptotic DNase when released from mitochondria. Nature.

[253] Asare PF, Roscioli E, Hurtado PR, Tran HB, Mah CY, Hodge S (2020). LC3-associated phagocytosis (LAP): a potentially influential mediator of efferocytosis-related tumor progression and aggressiveness. Front Oncol.

[254] Wang C, Youle RJ (2009). The role of mitochondria in apoptosis*. Annu Rev Genet.

[255] Soond SM, Carroll C, Townsend PA, Sayan E, Melino G, Behrmann I (2007). STAT1 regulates p73-mediated Bax gene expression. FEBS Lett.

[256] Cianfrocca R, Muscolini M, Marzano V, Annibaldi A, Marinari B, Levrero M (2008). RelA/NF-kappaB recruitment on the bax gene promoter antagonizes p73-dependent apoptosis in costimulated T cells. Cell Death Differ.

[257] Marabese M, Mazzoletti M, Vikhanskaya F, Broggini M (2008). HtrA2 enhances the apoptotic functions of p73 on bax. Cell Death Differ.

[258] Zhang H, Wu S, Xing D (2011). YAP accelerates Aβ(25–35)-induced apoptosis through upregulation of Bax expression by interaction with p73. Apoptosis.

[259] Yi L, Huang X, Guo F, Zhou Z, Chang M, Tang J (2016). Lipopolysaccharide induces human pulmonary micro-vascular endothelial apoptosis via the YAP signaling pathway. Front Cell Infect Microbiol.

[260] Melino G, Bernassola F, Ranalli M, Yee K, Zong WX, Corazzari M (2004). p73 Induces apoptosis via PUMA transactivation and Bax mitochondrial translocation. J Biol Chem.

[261] Ramadan S, Terrinoni A, Catani MV, Sayan AE, Knight RA, Mueller M (2005). p73 induces apoptosis by different mechanisms. Biochem Biophys Res Commun.

[262] Fricker M, Papadia S, Hardingham GE, Tolkovsky AM (2010). Implication of TAp73 in the p53-independent pathway of Puma induction and Puma-dependent apoptosis in primary cortical neurons. J Neurochem.

[263] Ming L, Sakaida T, Yue W, Jha A, Zhang L, Yu J (2008). Sp1 and p73 activate PUMA following serum starvation. Carcinogenesis.

[264] John K, Alla V, Meier C, Pützer BM (2011). GRAMD4 mimics p53 and mediates the apoptotic function of p73 at mitochondria. Cell Death Differ.

[265] Sasaki Y, Mita H, Toyota M, Ishida S, Morimoto I, Yamashita T (2003). Identification of the interleukin 4 receptor alpha gene as a direct target for p73. Cancer Res.

[266] Schuster A, Schilling T, De Laurenzi V, Koch AF, Seitz S, Staib F (2010). ΔNp73β is oncogenic in hepatocellular carcinoma by blocking apoptosis signaling via death receptors and mitochondria. Cell Cycle.

[267] Sayan AE, Sayan BS, Gogvadze V, Dinsdale D, Nyman U, Hansen TM (2008). P73 and caspase-cleaved p73 fragments localize to mitochondria and augment TRAIL-induced apoptosis. Oncogene.

[268] Yoon M-K, Kim B-Y, Lee J-Y, Ha J-H, Kim SA, Lee D-H (2018). Cytoplasmic pro-apoptotic function of the tumor suppressor p73 is mediated through a modified mode of recognition of the anti-apoptotic regulator Bcl-XL. J Biol Chem.

[269] Irwin M, Marin MC, Phillips AC, Seelan RS, Smith DI, Liu W (2000). Role for the p53 homologue p73 in E2F-1-induced apoptosis. Nature.

[270] Stiewe T, Pützer BM (2000). Role of the p53-homologue p73 in E2F1-induced apoptosis. Nat Genet.

[271] Tophkhane C, Yang S-H, Jiang Y, Ma Z, Subramaniam D, Anant S, et al. p53 inactivation upregulates p73 expression through E2F-1 mediated transcription. PLoS ONE. 2012;7(8):e43564. 10.1371/journal.pone.0043564 PMC3431388 22952705

[272] DeYoung MP, Johannessen CM, Leong C-O, Faquin W, Rocco JW, Ellisen LW (2006). Tumor-specific p73 up-regulation mediates p63 dependence in squamous cell carcinoma. Cancer Res.

[273] Rocco JW, Leong C-O, Kuperwasser N, DeYoung MP, Ellisen LW (2006). p63 mediates survival in squamous cell carcinoma by suppression of p73-dependent apoptosis. Cancer Cell.

[274] Leong C-O, Vidnovic N, DeYoung MP, Sgroi D, Ellisen LW (2007). The p63/p73 network mediates chemosensitivity to cisplatin in a biologically defined subset of primary breast cancers. J Clin Invest.

[275] Liu T, Roh SE, Woo JA, Ryu H, Kang DE. Cooperative role of RanBP9 and P73 in mitochondria-mediated apoptosis. Cell Death Dis. 2013;4:e476. 10.1038/cddis.2012.203 PMC3563991 23348590

[276] Costanzo A, Merlo P, Pediconi N, Fulco M, Sartorelli V, Cole PA (2002). DNA damage-dependent acetylation of p73 dictates the selective activation of apoptotic target genes. Mol Cell.

[277] He H, Wang C, Dai Q, Li F, Bergholz J, Li Z (2016). p53 and p73 regulate apoptosis but not cell-cycle progression in mouse embryonic stem cells upon DNA damage and differentiation. Stem Cell Rep.

[278] Agami R, Blandino G, Oren M, Shaul Y (1999). Interaction of c-Abl and p73alpha and their collaboration to induce apoptosis. Nature.

[279] Li Q, Huang Z, Gao M, Cao W, Xiao Q, Luo H, et al. Blockade of Y177 and nuclear translocation of Bcr-Abl inhibits proliferation and promotes apoptosis in chronic myeloid leukemia cells. Int J Mol Sci. 2017;18(3). 10.3390/ijms18030537 PMC5372553 28257089

[280] Tanwar K, Pati U. Inhibition of apoptosis via CHIP-mediated proteasomal degradation of TAp73α. J Cell Biochem. 2019. 10.1002/jcb.28386 30714204

[281] Deponte M (2013). Glutathione catalysis and the reaction mechanisms of glutathione-dependent enzymes. Biochim Biophys Acta.

[282] Geiger A (1935). Rôle of glutathione in anaerobic tissue glycolysis. Biochem J.

[283] Li Y, Cao Y, Xiao J, Shang J, Tan Q, Ping F (2020). Inhibitor of apoptosis-stimulating protein of p53 inhibits ferroptosis and alleviates intestinal ischemia/reperfusion-induced acute lung injury. Cell Death Differ.

[284] Vance JE (2020). Inter-organelle membrane contact sites: implications for lipid metabolism. Biol Direct.

[285] Celardo I, Melino G, Amelio I (2020). Commensal microbes and p53 in cancer progression. Biol Direct.

[286] Schmelz K, Wagner M, Dörken B, Tamm I (2005). 5-Aza-2’-deoxycytidine induces p21WAF expression by demethylation of p73 leading to p53-independent apoptosis in myeloid leukemia. Int J Cancer.

[287] Muscolini M, Cianfrocca R, Sajeva A, Mozzetti S, Ferrandina G, Costanzo A (2008). Trichostatin A up-regulates p73 and induces Bax-dependent apoptosis in cisplatin-resistant ovarian cancer cells. Mol Cancer Ther.

[288] Warburg O, Wind F, Negelein E (1926). Über den stoffwechsel von tumoren im körper. Klin Wochenschr.

[289] Liberti MV, Locasale JW (2016). The warburg effect: how does it benefit cancer cells?. Trends Biochem Sci.

[290] Agostini M, Annicchiarico-Petruzzelli M, Melino G, Rufini A (2016). Metabolic pathways regulated by TAp73 in response to oxidative stress. Oncotarget.

[291] Dobon B, Montanucci L, Peretó J, Bertranpetit J, Laayouni H (2019). Gene connectivity and enzyme evolution in the human metabolic network. Biol Direct.

[292] Amelio I, Bertolo R, Bove P, Buonomo OC, Candi E, Chiocchi M (2020). Liquid biopsies and cancer omics. Cell Death Discov.

[293] He Z, Agostini M, Liu H, Melino G, Simon H-U (2015). p73 regulates basal and starvation-induced liver metabolism in vivo. Oncotarget.

[294] Amelio I, Antonov AA, Catani MV, Massoud R, Bernassola F, Knight RA (2014). TAp73 promotes anabolism. Oncotarget.

[295] Xu Y, Chen X (2006). Glyoxalase II, a detoxifying enzyme of glycolysis byproduct methylglyoxal and a target of p63 and p73, is a pro-survival factor of the p53 family. J Biol Chem.

[296] Sharif T, Dai C, Martell E, Ghassemi-Rad MS, Hanes MR, Murphy PJ (2019). TAp73 modifies metabolism and positively regulates growth of cancer stem-like cells in a redox-sensitive manner. Clin Cancer Res.

[297] Mullarky E, Cantley LC. Diverting glycolysis to combat oxidative stress. In: Nakao K, Minato N, Uemoto S, editors. Innovative medicine: basic research and development. Springer, Tokyo; 2015. 29787184

[298] Kuehne A, Emmert H, Soehle J, Winnefeld M, Fischer F, Wenck H (2015). Acute activation of oxidative pentose phosphate pathway as first-line response to oxidative stress in human skin cells. Mol Cell.

[299] Ben-Yoseph O, Boxer PA, Ross BD (1996). Assessment of the role of the glutathione and pentose phosphate pathways in the protection of primary cerebrocortical cultures from oxidative stress. J Neurochem.

[300] Ataullakhanov FI, Buravtsev VN, Zhabotinskiĩ AM, Norina SB, Pichugin AV (1981). Interaction of the Embden-Meyerhof pathway and hexose monophosphate shunt in erythrocytes. Biokhimiia.

[301] Pallucca R, Visconti S, Camoni L, Cesareni G, Melino S, Panni S, et al. Specificity of ε and non-ε isoforms of arabidopsis 14–3–3 proteins towards the H+-ATPase and other targets. PLoS ONE. 2014;9(6):e90764. 10.1371/journal.pone.0090764 PMC3946203 24603559

[302] Lamastra FR, De Angelis R, Antonucci A, Salvatori D, Prosposito P, Casalboni M (2014). Polymer composite random lasers based on diatom frustules as scatterers. RSC Adv.

[303] Mauretti A, Neri A, Kossover O, Seliktar D, Nardo PD, Melino S (2016). Design of a novel composite H2 S-releasing hydrogel for cardiac tissue repair. Macromol Biosci.

[304] Chaneton B, Hillmann P, Zheng L, Martin ACL, Maddocks ODK, Chokkathukalam A (2012). Serine is a natural ligand and allosteric activator of pyruvate kinase M2. Nature.

[305] Zahra K, Dey T, Ashish, Mishra SP, Pandey U. Pyruvate kinase M2 and cancer: the role of PKM2 in promoting tumorigenesis. Front Oncol. 2020;10:159. 10.3389/fonc.2020.00159 PMC7061896 32195169

[306] Wright WE, Piatyszek MA, Rainey WE, Byrd W, Shay JW (1996). Telomerase activity in human germline and embryonic tissues and cells. Dev Genet.

[307] Hayflick L (1965). The limited in vitro lifetime of human diploid cell strains. Exp Cell Res.

[308] Olovnikov AM (1971). Principle of marginotomy in template synthesis of polynucleotides. Dokl Akad Nauk SSSR.

[309] Olovnikov AM (1973). A theory of marginotomy. J Theor Biol.

[310] Shahbandi A, Rao SG, Anderson AY, Frey WD, Olayiwola JO, Ungerleider NA (2020). BH3 mimetics selectively eliminate chemotherapy-induced senescent cells and improve response in TP53 wild-type breast cancer. Cell Death Differ.

[311] Di Micco R, Sulli G, Dobreva M, Liontos M, Botrugno OA, Gargiulo G (2011). Interplay between oncogene-induced DNA damage response and heterochromatin in senescence and cancer. Nat Cell Biol.

[312] Sati S, Bonev B, Szabo Q, Jost D, Bensadoun P, Serra F (2020). 4D genome rewiring during oncogene-induced and replicative senescence. Mol Cell.

[313] Van Nguyen T, Puebla-Osorio N, Pang H, Dujka ME, Zhu C (2007). DNA damage-induced cellular senescence is sufficient to suppress tumorigenesis: a mouse model. J Exp Med.

[314] Davalli P, Mitic T, Caporali A, Lauriola A, D’Arca D (2016). ROS, cell senescence, and novel molecular mechanisms in aging and age-related diseases. Oxid Med Cell Longev.

[315] Macip S, Kosoy A, Lee SW, O’Connell MJ, Aaronson SA (2006). Oxidative stress induces a prolonged but reversible arrest in p53-null cancer cells, involving a Chk1-dependent G2 checkpoint. Oncogene.

[316] Sabelli R, Iorio E, De Martino A, Podo F, Ricci A, Viticchiè G (2008). Rhodanese-thioredoxin system and allyl sulfur compounds. FEBS J.

[317] Nepravishta R, Sabelli R, Iorio E, Micheli L, Paci M, Melino S (2012). Oxidative species and S-glutathionyl conjugates in the apoptosis induction by allyl thiosulfate. FEBS J.

[318] Tavana O, Benjamin CL, Puebla-Osorio N, Sang M, Ullrich SE, Ananthaswamy HN (2010). Absence of p53-dependent apoptosis leads to UV radiation hypersensitivity, enhanced immunosuppression and cellular senescence. Cell Cycle.

[319] Zhong G, Qin S, Townsend D, Schulte BA, Tew KD, Wang GY (2019). Oxidative stress induces senescence in breast cancer stem cells. Biochem Biophys Res Commun.

[320] Banerjee K, Mandal M (2015). Oxidative stress triggered by naturally occurring flavone apigenin results in senescence and chemotherapeutic effect in human colorectal cancer cells. Redox Biol.

[321] ICGC/TCGA Pan-Cancer Analysis of Whole Genomes Consortium (2020). Pan-cancer analysis of whole genomes. Nature.

[322] Chan KT, Blake S, Zhu H, Kang J, Trigos AS, Madhamshettiwar PB (2020). A functional genetic screen defines the AKT-induced senescence signaling network. Cell Death Differ.

[323] Shao J, Lu J, Zhu W, Yu H, Jing X, Wang Y-L (2019). Derepression of LOXL4 inhibits liver cancer growth by reactivating compromised p53. Cell Death Differ.

[324] Serrano M, Lin AW, McCurrach ME, Beach D, Lowe SW (1997). Oncogenic ras provokes premature cell senescence associated with accumulation of p53 and p16INK4a. Cell.

[325] Takaoka M, Harada H, Deramaudt TB, Oyama K, Andl CD, Johnstone CN (2004). Ha-Ras(G12V) induces senescence in primary and immortalized human esophageal keratinocytes with p53 dysfunction. Oncogene.

[326] Lin AW, Lowe SW (2001). Oncogenic ras activates the ARF-p53 pathway to suppress epithelial cell transformation. Proc Natl Acad Sci USA.

[327] Li T, Kon N, Jiang L, Tan M, Ludwig T, Zhao Y (2012). Tumor suppression in the absence of p53-mediated cell-cycle arrest, apoptosis, and senescence. Cell.

[328] Marini A, Rotblat B, Sbarrato T, Niklison-Chirou MV, Knight JRP, Dudek K (2018). TAp73 contributes to the oxidative stress response by regulating protein synthesis. Proc Natl Acad Sci USA.

[329] Yao Y, Bellon M, Shelton SN, Nicot C (2012). Tumor suppressors p53, p63TAα, p63TAy, p73α, and p73β use distinct pathways to repress telomerase expression. J Biol Chem.

[330] Velletri T, Romeo F, Tucci P, Peschiaroli A, Annicchiarico-Petruzzelli M, Niklison-Chirou MV (2013). GLS2 is transcriptionally regulated by p73 and contributes to neuronal differentiation. Cell Cycle.

[331] Hu W, Zhang C, Wu R, Sun Y, Levine A, Feng Z (2010). Glutaminase 2, a novel p53 target gene regulating energy metabolism and antioxidant function. Proc Natl Acad Sci USA.

[332] Zhang J, Kong X, Zhang Y, Sun W, Wang J, Chen M (2020). FDXR regulates TP73 tumor suppressor via IRP2 to modulate aging and tumor suppression. J Pathol.

[333] Fujita K. p53 Isoforms in cellular senescence- and ageing-associated biological and physiological functions. Int J Mol Sci. 2019;20(23). 10.3390/ijms20236023 PMC6928910 31795382

[334] Horikawa I, Fujita K, Jenkins LMM, Hiyoshi Y, Mondal AM, Vojtesek B (2014). Autophagic degradation of the inhibitory p53 isoform Δ133p53α as a regulatory mechanism for p53-mediated senescence. Nat Commun.

[335] Qian Y, Chen X (2013). Senescence regulation by the p53 protein family. Methods Mol Biol.

[336] Mijit M, Caracciolo V, Melillo A, Amicarelli F, Giordano A. Role of p53 in the regulation of cellular senescence. Biomolecules. 2020;10(3). 10.3390/biom10030420 PMC7175209 32182711

[337] Lee HC, Kang D, Han N, Lee Y, Hwang HJ, Lee S-B (2020). A novel long noncoding RNA Linc-ASEN represses cellular senescence through multileveled reduction of p21 expression. Cell Death Differ.

[338] Lin C, Li H, Liu J, Hu Q, Zhang S, Zhang N (2020). Arginine hypomethylation-mediated proteasomal degradation of histone H4-an early biomarker of cellular senescence. Cell Death Differ.

[339] Aird KM, Zhang R. Metabolic alterations accompanying oncogene-induced senescence. Mol Cell Oncol. 2014;1(3):e963481. 10.4161/23723548.2014.963481 PMC4904889 27308349

[340] Aird KM, Zhang G, Li H, Tu Z, Bitler BG, Garipov A (2013). Suppression of nucleotide metabolism underlies the establishment and maintenance of oncogene-induced senescence. Cell Rep.

[341] Fatt MP, Cancino GI, Miller FD, Kaplan DR (2014). p63 and p73 coordinate p53 function to determine the balance between survival, cell death, and senescence in adult neural precursor cells. Cell Death Differ.

[342] Amir M, Khan P, Queen A, Dohare R, Alajmi MF, Hussain A, et al. Structural features of nucleoprotein CST/shelterin complex involved in the telomere maintenance and its association with disease mutations. Cells. 2020;9(2). 10.3390/cells9020359 PMC7072152 32033110

[343] Liu G, Chen X (2002). The ferredoxin reductase gene is regulated by the p53 family and sensitizes cells to oxidative stress-induced apoptosis. Oncogene.

[344] Zhang Y, Qian Y, Zhang J, Yan W, Jung Y-S, Chen M (2017). Ferredoxin reductase is critical for p53-dependent tumor suppression via iron regulatory protein 2. Genes Dev.

[345] Liu L, Wang G, Wang L, Yu C, Li M, Song S (2020). Computational identification and characterization of glioma candidate biomarkers through multi-omics integrative profiling. Biol Direct.

[346] Kim SY, Jeong H-H, Kim J, Moon J-H, Sohn K-A (2019). Robust pathway-based multi-omics data integration using directed random walks for survival prediction in multiple cancer studies. Biol Direct.

